# Targeting the Crosstalk Between Metabolism and Chronic Inflammation: In Silico Multitargeting Drug Design Approach for Cardiometabolic Syndrome

**DOI:** 10.3390/biomedicines14061213

**Published:** 2026-05-27

**Authors:** Errikos Petsas, Gerasimos Siasos, Thomas Mavromoustakos, Christos T. Chasapis

**Affiliations:** 1 Laboratory of Organic Chemistry, Department of Chemistry, National and Kapodistrian University of Athens, 15771 Athens, Greece; errpets@chem.uoa.gr (E.P.); tmavrom@chem.uoa.gr (T.M.); 2 3rd Department of Cardiology, Thoracic Diseases General Hospital Sotiria, Medical School, National and Kapodistrian University of Athens, 11527 Athens, Greece; gsiasos@med.uoa.gr

**Keywords:** multitarget drug design, medicinal chemistry, molecular docking, molecular dynamics, cardiometabolic syndrome, type 2 diabetes, cholesterol, obesity, inflammation, in silico ADMET

## Abstract

**Βackround/Objectives:** The rising global burden of cardiometabolic disorders and chronic low-grade inflammation underscores the need for therapies capable of modulating multiple interconnected pathways. **Methods:** In this work, a ligand-based virtual screening campaign centered on a previously reported scaffold (compound 1a) was combined with molecular docking, 200 ns molecular dynamics simulations and ADMET prediction to identify and prioritize small-molecule multitarget candidates against PCSK9, GLP1R, FGFR1, GIPR, NF-κB and NLRP3. **Results:** Among the screened analogs, D4Z emerged as the most balanced lead, displaying consistently favorable binding profiles, stable interactions within functionally relevant pockets and a drug-like physicochemical and pharmacokinetic profile with high predicted oral absorption. **Conclusions:** Although these findings remain purely computational, they support D4Z as a prioritized multitarget lead for synthesis and experimental validation and illustrate the potential of rational multitarget design for addressing the cardiometabolic–inflammatory axis.

## 1. Introduction

The modern pharmacological landscape is increasingly shifting from the traditional “one drug, one target” paradigm toward multitargeting drug design, particularly for the management of complex, chronic conditions [1,2]. Cardiometabolic diseases and systemic inflammatory disorders are inherently multifactorial, involving an intricate crosstalk between lipid metabolism, glucose regulation, and innate immune responses [3]. Therefore, developing a single chemical entity capable of simultaneously modulating multiple biological pathways offers potential advantages, including enhanced therapeutic efficacy, reduced risk of drug–drug interactions, and improved patient compliance [4].

Among these interconnected pathologies, cardiometabolic syndrome exemplifies the tight interplay between dyslipidemia, insulin resistance, obesity, and low-grade chronic inflammation. In this setting, disturbances in lipid metabolism and glucose homeostasis perpetuate inflammatory signaling, while inflammation further aggravates atherosclerosis and metabolic dysfunction [5]. As a result, single-target interventions often provide incomplete benefit and necessitate combination regimens, increasing pill burden and adverse event risk. Rationally designed multitarget small molecules therefore offer an attractive opportunity to simultaneously address metabolic and inflammatory pathways within one pharmacokinetically coherent scaffold.

The therapeutic targets selected in this study reflect key regulatory hubs within this cardiometabolic–inflammatory axis. Proprotein convertase subtilisin/kexin type 9 (PCSK9) is a critical modulator of low-density lipoprotein receptor (LDLR) turnover and thus a central regulator of plasma LDL-cholesterol levels and atherosclerotic risk [6,7]. Incretin receptors, namely the glucagon-like peptide-1 receptor (GLP1R) [8] and the glucose-dependent insulinotropic polypeptide receptor (GIPR), have emerged as clinically validated targets for the treatment of type 2 diabetes and obesity, with dual or multiagonists showing superior metabolic outcomes compared to single-receptor agonists [9,10]. Fibroblast growth factor receptor 1 (FGFR1), through its interaction with FGF21, is implicated in energy expenditure, glucose utilization, and lipid metabolism, providing an additional point of metabolic intervention [11]. On the inflammatory side, the transcription factor NF-κB orchestrates the expression of numerous pro-inflammatory mediators, while the NLRP3 inflammasome controls the maturation and release of interleukin-1β and interleukin-18, acting as a major driver of chronic low-grade inflammation and insulin resistance [12,13].

Collectively, these six proteins define a mechanistically coherent framework connecting cholesterol homeostasis, incretin signaling, metabolic control, and innate immune activation. From a drug design perspective, the challenge is to engineer a single small molecule capable of achieving high-affinity and topologically compatible engagement across these heterogeneous binding environments without compromising drug-likeness. To address this challenge, we adopted a rational multitarget design strategy grounded on three fundamental pharmacophoric pillars: (i) incorporation of aromatic moieties to facilitate π–π stacking and edge-to-face contacts with key residues, (ii) utilization of hydrophobic substituents to occupy nonpolar regions and reinforce van der Waals interactions, and (iii) strategic positioning of polar functionalities to establish directional hydrogen-bonding networks and, where appropriate, salt bridges with charged residues.

To navigate the complex distribution of cationic and anionic residues across the six receptor pockets, a modular ligand architecture was implemented, featuring a rigid heteroaromatic core serving as a central anchor. This core provides a structurally defined platform for context-dependent formation of hydrogen bonds and electrostatic contacts, while peripheral substituents modulate lipophilicity and polarity in line with drug-likeness constraints. Particular attention was paid to maintaining physicochemical properties compatible with oral administration, including a molecular weight in the low-to-medium range, a topological polar surface area below approximately 140 Å^2^, and compliance with established rules of drug-likeness, so that the resulting hybrid would remain both pharmacokinetically viable and synthetically accessible.

**Figure 1:**
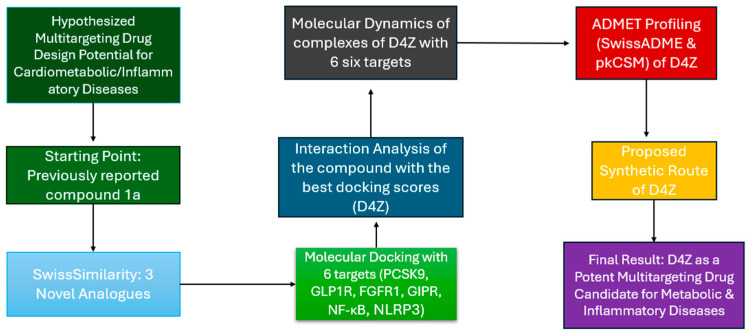
Integrated in silico workflow for the discovery of D4Z. The pipeline illustrates the transition from the initial lead scaffold 1a [14] to the identification, docking and dynamic validation across six therapeutic targets, ADMET and synthetic assessment of the final multitargeting candidate.

Within this design framework, a SwissSimilarity-guided analog search was performed using the previously reported lead scaffold (compound 1a) [14] as the starting point, leading to the identification of D4Z as the most balanced in silico multitarget candidate for further investigation. The subsequent optimization and prioritization of D4Z were supported by an integrated in silico workflow that combined ligand-based design principles, molecular docking, molecular dynamics (MD) simulations and ADMET profiling. This approach enabled iterative refinement of the ligand architecture to enhance binding complementarity across PCSK9, GLP1R, FGFR1, GIPR, NF-κB and NLRP3 while maintaining a balanced physicochemical profile. The overall discovery pipeline, from initial scaffold evaluation and analogue screening to the multi-level computational characterization of D4Z, is summarized in Figure 1.

In this work, the in silico design and computational characterization of D4Z are described as a multitarget small-molecule modulator targeting key cardiometabolic and inflammatory pathways. By integrating molecular docking, 200 ns MD simulations, and ADMET prediction tools, we aim to provide a mechanistically informed, computational proof-of-concept that such a single chemotype can achieve concurrent engagement of metabolic and inflammatory targets.

## 2. Materials and Methods

### 2.1. SwissSimilarity Ligand Search

An initial set of analogs of the lead scaffold 1a was generated using the SwissSimilarity web platform, yielding the compounds D4Z, 17X and K4F. The search for novel analogues was prompted by the weak docking performance of the original scaffold 1a across several of the six therapeutic targets. To identify candidates with superior multitarget potential, ligand-based virtual screening was performed against the “Ligands present in the PDB (LigandExpo)” collection using the Combined screening mode of SwissSimilarity. This approach integrates 2D FP2 fingerprint similarity and fast 3D Electroshape-5D similarity into a consensus score, allowing prioritization of analogues embedded in experimentally validated bioactive chemical space. Under these conditions, 17X, K4F and D4Z were ranked among the top hits, with combined similarity scores of 0.207, 0.199 and 0.187, respectively [15].

### 2.2. Protein Target Selection and Preparation

A panel of six protein targets was selected to represent key biological pathways involved in metabolic regulation, lipid homeostasis, and inflammation. The selected targets included PCSK9 (PDB ID: 2P4E), GLP1R (PDB ID: 6X18), FGFR1 (PDB ID: 4RWI), GIPR (PDB ID: 7RBT), NF-κB (PDB ID: 4Q3J), and NLRP3 (PDB ID: 5IRM). All protein structures were obtained from the Protein Data Bank (PDB) and processed using the Protein Preparation Wizard in Schrödinger Maestro Suite 2021-2. Preparation steps included assignment of bond orders, addition of hydrogen atoms, optimization of protonation states at physiological pH (7.4), refinement of the hydrogen bonding network, and restrained energy minimization using the OPLS4 force field [16].

### 2.3. Ligand Preparation and Geometry Optimization

Ligand preparation and geometry optimization were carried out in Maestro using LigPrep and MacroModel. Ionization states were generated with Epik (including metal-binding and original states) to reflect physiological conditions (pH 7.4) [15]. To ensure reliable 3D geometries, the structure was subjected to energy minimization using MacroModel under the OPLS4 force field. Minimization was performed using a conjugate gradient approach with a convergence threshold of 0.01 kcal/mol, ensuring the ligand reached its conformational state prior to docking simulations [17].

### 2.4. Molecular Docking Simulations

Induced-fit docking was performed in Schrödinger Maestro 2021-2 using the Extra Precision (XP) protocol, generating up to 10 poses per ligand for each target (chosen protein preparation constrained refinement and trim side chains based on Β-factor automatically). The actual Schrödinger Induced Fit Docking (IFD) workflow was adopted, where the initial docking settings generated up to 20 starting poses per ligand using a van der Waals radii scaling factor of 0.70 for the receptor and 0.50 for the ligand to accommodate conformational flexibility. Receptor grids were generated around the established active sites of each target, guided by co-crystallized ligands and key catalytic residues. Subsequently, prime refinement and side-chain optimization were executed using the Prime module for all amino acid residues located within a 5.0 Å radius of the docked ligand poses. Finally, the refined complexes were subjected to Glide redocking in Extra Precision (XP) mode, and the scoring criteria were evaluated using the comprehensive IFD Score to select the top 10 retained poses. Docking was applied consistently across all targets to ensure comparability of binding scores [18]. Interaction patterns were analyzed using the Ligand Interaction Diagram module to identify specific hydrogen bonds, hydrophobic contacts, and π-π stacking interactions [19].

Visual inspection and high-resolution rendering of the protein-ligand complexes were performed using UCSF Chimera (version 1.19) [20]. The software was utilized to generate molecular surfaces for both the established catalytic pockets (defined in yellow) and the docking-derived binding sites of compound D4Z (defined in blue). Alignment and overlap analysis were conducted to qualitatively assess the degree of structural complementarity between the lead candidate and the functionally critical domains of the six therapeutic targets. Final graphical representations, including stick and surface models, were exported as high-resolution images for comparative structural analysis.

### 2.5. Molecular Dynamics Simulations

To evaluate the temporal stability of the D4Z–protein complexes, molecular dynamics (MD) simulations were performed for 200 ns using the Desmond module in Maestro- Schrödinger 2021-2. For membrane-associated targets (GLP1R, GIPR), the complexes were embedded in a pre-equilibrated POPC phospholipid bilayer, simulated under an NPγT ensemble at 300 K with TIP3P solvation and 1 bar pressure, whereas PCSK9, NLRP3, NF-κB and FGFR1 were solvated in an explicit TIP3P water box, neutralized with counterions, and simulated under an NPT ensemble at 310 K and 1 bar. Trajectory analysis focused on backbone Cα root-mean-square fluctuation (RMSF) profiles and ligand RMSF values, protein RMSD backbone Cα, ligand RMSD, ligand–protein contact persistence, hydrogen-bond occupancy, radius of gyration to monitor conformational stability, binding persistence and local flexibility within the binding pockets [16,21].

### 2.6. In Silico ADMET Profiling

The drug-likeness and pharmacokinetic profile of D4Z were assessed using SwissADME and pkCSM. SwissADME was utilized to calculate physicochemical descriptors, Lipinski’s “Rule of Five” compliance, and GI absorption (detailed SwissADME prediction outputs are provided in the Supplementary Materials Figure S4) [22]. pkCSM provided quantitative predictions for distribution (BBB/CNS permeability), metabolism (CYP450 inhibition), and toxicity endpoints (AMES toxicity, hERG inhibition, and hepatotoxicity), ensuring an integrated evaluation of the compound’s safety and oral bioavailability [23].

### 2.7. Synthetic Route Design

The synthetic feasibility of D4Z was explored using the Reaxys medicinal chemistry database. A proposed hypothetical retrosynthetic analysis was performed to identify commercially available starting materials and optimize reaction conditions. The proposed hypothetical route prioritized high atom economy to ensure the compound’s accessibility for future experimental validation.

## 3. Results

### 3.1. Molecular Docking Results

The molecular docking simulations, performed using the Maestro Schrödinger 2021-2 suite, provided initial insights into the binding scores and orientation of the investigated compounds within the active sites of the six therapeutic targets. The docking scores are summarized in Table 1.

To evaluate the docking protocol and put the scores of D4Z into perspective, we compared its performance against known small-molecule inhibitors for each target (Table 1). The predicted scores show that D4Z matches or even exceeds the performance of these reference ligands. Specifically, in the PCSK9 pocket, D4Z scored −8.780 kcal/mol, outperforming SBC-115076 (−7.265 kcal/mol). For the GLP1R (−10.280 kcal/mol) and GIPR (−10.212 kcal/mol) orthosteric sites, D4Z showed better binding scores than danuglipron (−9.504 kcal/mol) and MK-0893 (−9.770 kcal/mol). In the FGFR1 kinase domain, its score (−10.432 kcal/mol) was practically identical to that of the approved drug erdafitinib ($−10.401 kcal/mol). Finally, D4Z also showed strong complementarity with the inflammatory targets, outscoring parthenolide on NF-κB (−8.115 vs. −7.102 kcal/mol) and MCC-950 on NLRP3 (−10.620 vs. −9.430 kcal/mol).

The comparative assessment of the molecular docking scores reveals that compound D4Z displays the most balanced high-affinity profile across all six targets, whereas 17X, despite showing slightly lower docking scores for GIPR and NLRP3, suffers from poor ADMET properties. With binding scores ranging from −8.115 to −10.620 kcal/mol, D4Z significantly outperformed the initial lead compound 1a, which displayed the weakest affinity across all proteins. The consistently high performance of D4Z, particularly within receptors governing metabolism and inflammation, supports the scaffold prioritization strategy performed via SwissSimilarity and establishes it as a superior lead candidate for further development.

In terms of target-specific efficacy, D4Z demonstrated complementarity with the incretin receptors GLP1R (−10.280 kcal/mol) and GIPR (−10.212 kcal/mol), suggesting strong potential for the management of type 2 diabetes and obesity-related weight loss. Concurrently, its high scores against NLRP3 (−10.620 kcal/mol) and NF-κB (−8.115 kcal/mol) highlight its capacity to modulate critical inflammatory pathways. While compound 17X showed marginally higher affinity in specific instances, such as with NLRP3 (−12.270 kcal/mol) and GIPR (−11.320 kcal/mol), D4Z is distinguished by its balanced affinity across all six targets, facilitating a multi-target approach for cardioprotection and cholesterol regulation via PCSK9 inhibition.

The thermodynamic stability suggested by these docking scores indicates that D4Z possesses the necessary structural features to effectively occupy the diverse active sites of the studied proteins. Its ability to maintain good binding scores across functionally distinct targets underscores its potential as a versatile pharmacological agent. This broad-spectrum binding profile suggests that D4Z emerges as a structurally promising multitarget lead candidate, offering a unified therapeutic strategy for addressing interrelated metabolic and inflammatory syndromes.

### 3.2. Analysis of 2D Ligand Interaction Diagrams of D4Z with the Six Targets

To provide a deeper understanding of the molecular recognition patterns between the lead compound D4Z and the six therapeutic targets, a detailed analysis of the 2D ligand interaction diagrams was conducted. While the docking scores offer a quantitative measure of binding score, these diagrams provide complementary qualitative insight into the underlying interaction networks. The interaction profile of D4Z was evaluated across all six receptors: PCSK9, GLP1R, FGFR1, GIPR, NF-κB, and NLRP3. Examination of the 2D interaction diagrams allows the identification of key amino acid residues that contribute to the stability of the complexes observed in the docking results and supports the structural basis of the proposed multitarget pharmacological profile of D4Z.

**Figure 2:**
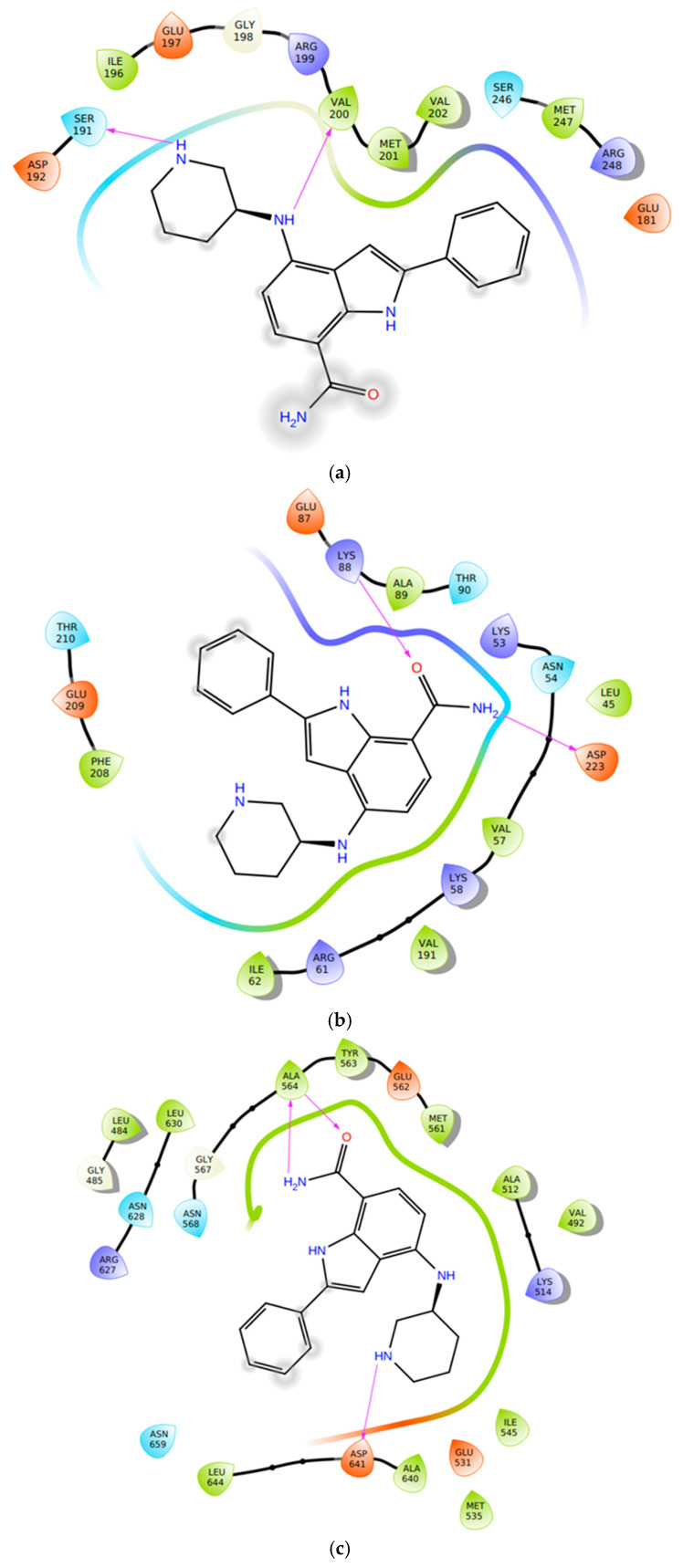

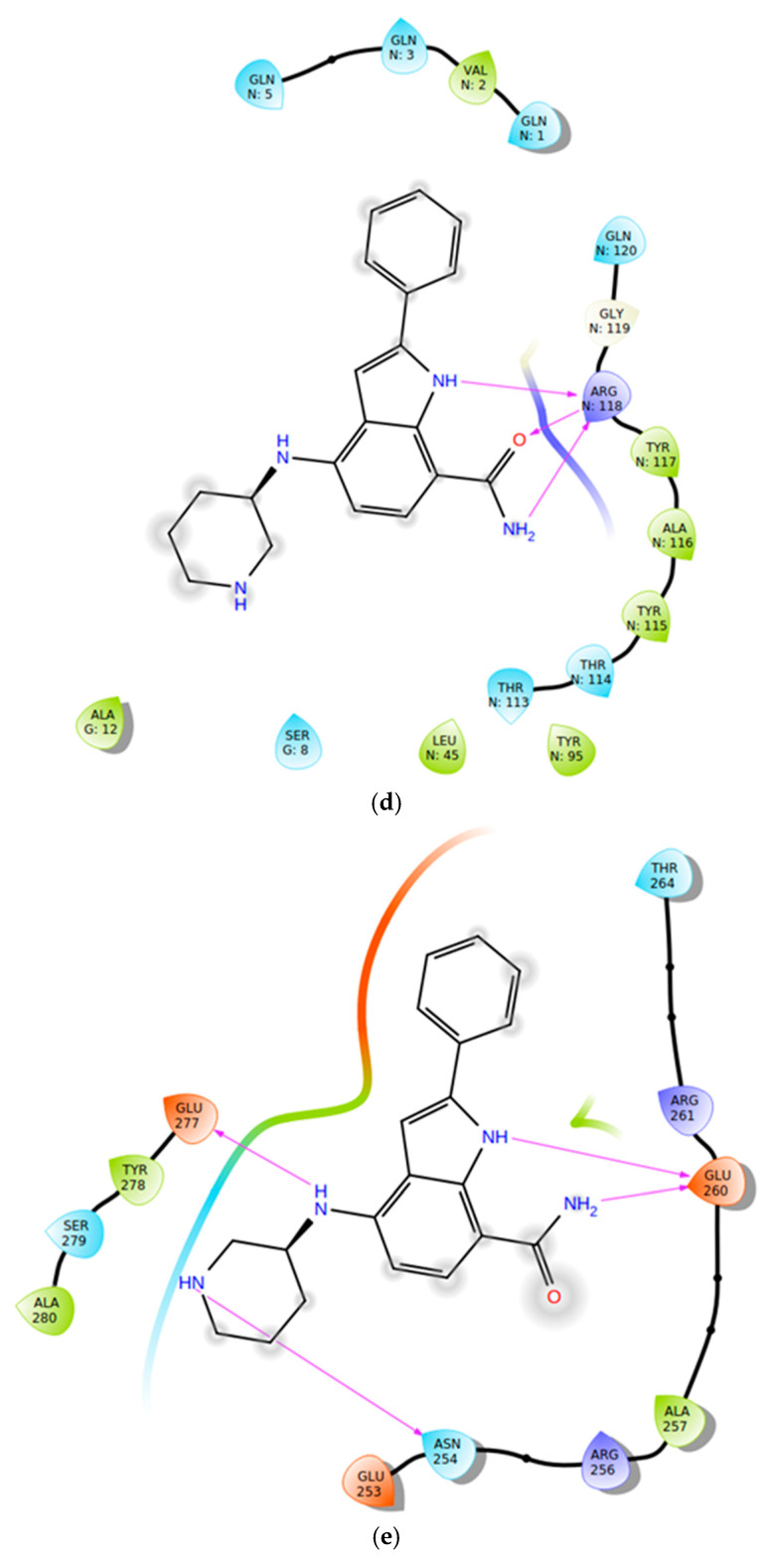

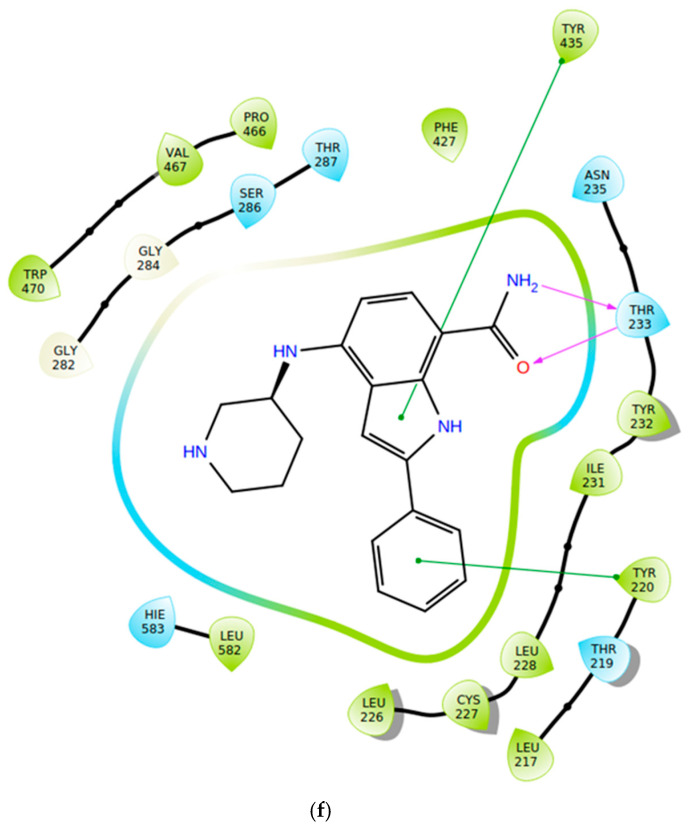
(**a**): Two-dimensional (2D) ligand interaction diagram of the lead candidate D4Z within the binding pocket of Human PCSK9 (PDB ID: 2P4E). The illustration delineates the specific molecular recognition motifs, highlighting directional hydrogen bonding (purple arrows) and a complementary network of hydrophobic and electrostatic interactions with key active site residues. (**b**): Two-dimensional (2D) ligand interaction diagram of compound D4Z within the binding pocket of GLP1R (PDB ID: 6X18). The complex achieved a docking score of −10.280 kcal/mol, characterized by essential hydrogen bonds (purple arrows) with Lys88 and Asp223, alongside a broad hydrophobic interaction network. (**c**): Two-dimensional (2D) ligand interaction diagram of compound D4Z within the binding pocket of FGFR1 (PDB ID: 4RWI). The complex, which achieved a docking score of −10.432 kcal/mol, is stabilized by essential hydrogen bonds (purple arrows) with Ala564 and Asp641, along with a dense network of hydrophobic interactions. (**d**): Two-dimensional (2D) ligand interaction diagram of compound D4Z within the binding pocket of GIPR (PDB ID: 7RBT). The complex, which achieved a docking score of −10.212 kcal/mol, is characterized by a critical hydrogen bonding network (purple arrows) with Arg118, alongside stabilizing hydrophobic contacts with the surrounding receptor residues. (**e**): Two-dimensional (2D) ligand interaction diagram of compound D4Z within the binding pocket of NF-κB (PDB ID: 4Q3J). The complex, which achieved a docking score of −8.115 kcal/mol, is stabilized by a network of four hydrogen bonds (purple arrows) with Glu260, Glu277, and Asn254, alongside complementary hydrophobic and electrostatic interactions. (**f**): Two-dimensional (2D) ligand interaction diagram of compound D4Z within the nucleotide-binding domain of the NLRP3 inflammasome (PDB ID: 5IRM). The diagram illustrates the displacement of the native ADP by D4Z, stabilized by bifurcated hydrogen bonds (purple arrows), π-π stacking interactions (green lines), and an extensive hydrophobic envelope.

The in silico evaluation of compound D4Z against the PCSK9 catalytic domain (PDB ID: 2P4E) yielded a docking score of −8.780 kcal/mol. As indicated by the interaction diagram, the protein–ligand complex is primarily stabilized by a dual hydrogen-bonding network. Specifically, the nitrogen atom within the piperidine ring functions as a hydrogen bond donor to the side chain of Ser191, while the secondary amine of the linker forms a second hydrogen bond with the backbone of Val200, anchoring the ligand scaffold within the receptor’s binding groove (Figure 2a). Complementing these polar contacts, the aromatic system of D4Z is accommodated within a lipophilic pocket formed by Ile196, Val200, Met201, Val202, and Met247, whereas Asp192, Glu197, Gly198, Arg199, Ser246, Arg248, and Glu181 define a polar and charged environment around the binding site. The overall interaction pattern suggests a high degree of structural complementarity consistent with inhibition of PCSK9-mediated pathways in cholesterol regulation.

Molecular docking simulations for D4Z against GLP1R (PDB ID: 6X18) indicate a strong binding score, with a docking score of −10.280 kcal/mol. As shown in Figure 2b, the ligand is embedded within the receptor’s binding pocket and stabilized by two principal hydrogen bonds involving its amide group: the carbonyl oxygen of D4Z accepts a hydrogen bond from the side chain of Lys88, whereas the terminal amine donates a hydrogen bond to the carboxylate group of Asp223. The core scaffold and phenyl substituent are further accommodated within a lipophilic environment formed by Leu45, Val57, Ile62, Ala89, and Phe208, while Glu87, Arg61, Lys53, Lys58, and Asn54 contribute additional polar and charged contacts. This interaction pattern is consistent with occupancy of a key regulatory region of GLP1R, supporting the potential D4Z suggests a potential for modulating incretin receptor conformations.

Docking analysis of D4Z against the FGFR1 kinase domain (PDB ID: 4RWI) revealed a docking score of −10.432 kcal/mol, indicative of a fit within the ATP-binding pocket. The ligand is anchored by three key hydrogen bonds involving both the amide substituent and the piperidine ring: the amide nitrogen donates a hydrogen bond to the backbone carbonyl of Ala564, the amide oxygen accepts a second hydrogen bond from the same residue, and the piperidine nitrogen forms a third hydrogen bond with the carboxylate group of Asp641 (Figure 2c). Beyond these polar interactions, the aromatic core of D4Z is positioned within a lipophilic cavity lined by Leu484, Val492, Ala512, Lys514, Met561, Tyr563, Leu630, and Leu644, whose hydrophobic and van der Waals contacts contribute to the overall binding energy. These findings support the notion that D4Z can effectively occupy the FGFR1 ATP pocket and which is consistent with a hypothetical modulation of FGF-driven downstream pathways.

Molecular docking simulations for D4Z against GIPR (PDB ID: 7RBT) yielded a docking score of −10.212 kcal/mol, consistent with a strong interaction at the receptor binding site. As depicted in Figure 2d, the complex is dominated by a well-defined interaction hub centered on Arg118 (Chain N). The amide group of D4Z acts as a dual anchor, with its carbonyl oxygen serving as a hydrogen bond acceptor and its terminal amine as a donor to the side chain of Arg118, while the indole nitrogen forms an additional hydrogen bond with the same residue. This triple-point polar attachment stabilizes the ligand core, which is further supported by hydrophobic and van der Waals contacts with Val2, Leu45, Tyr95, Ala116, and Tyr117 (Chain N), as well as Ala12 and Ser8 (Chain G). The targeting of the N-terminal extracellular domain of GIPR, which plays a central role in native incretin recognition27, suggests that D4Z may act as a high-affinity modulator of GIPR signaling.

Docking of D4Z into the NF-κB binding site (PDB ID: 4Q3J) produced a docking score of −8.115 kcal/mol. The interaction diagram (Figure 2e) indicates that the complex is stabilized by a dense polar network: the indole nitrogen and the primary amine of the amide group donate hydrogen bonds to the side chain of Glu260, the secondary amine of the linker donates a hydrogen bond to Glu277, and the piperidine nitrogen forms a hydrogen bond with Asn254. Additional stabilization arises from contacts with Tyr278, Ala280, Ala257, and Arg256, which contribute hydrophobic and electrostatic interactions. The presence of several glutamic acid residues (Glu260, Glu277, Glu253) around the ligand underscores the highly polar nature of this binding region and supports a potential role of D4Z in modulating NF-κB–mediated inflammatory signaling.

The computational investigation of D4Z against the NLRP3 inflammasome NACHT domain (PDB ID: 5IRM) revealed a docking score of −10.620 kcal/mol, representing the most preferable interaction across the studied panel. In this model, D4Z occupies the conserved ATP/ADP-binding pocket and competes with the native nucleotide. The primary stabilization arises from hydrogen bonds between the amide substituent of D4Z and Thr233, where the ligand functions both as a donor (terminal amine) and an acceptor (carbonyl oxygen). Additional stabilization is provided by π–π stacking interactions between the aromatic rings of D4Z and the side chains of Tyr435 and Tyr220 (Figure 2f), as well as an extensive hydrophobic environment formed by Leu217, Thr219, Leu226, Cys227, Leu228, Ile231, Tyr232, Asn235, Phe427, Pro466, Val467, Trp470, Gly282, Gly284, Ser286, Thr287, Leu582, and His583. These contacts ensure that D4Z remains tightly anchored within the nucleotide-binding site and are consistent with a potential inhibitory effect on NLRP3 activation.

### 3.3. Comparison of D4Z Binding with Defined Orthosteric Pockets

To further assess the biological relevance of the docking poses, a comparative analysis was performed between the binding site of compound D4Z and the established orthosteric pockets of the six therapeutic targets defined in a previous study14. The catalytic residues and active-site coordinates were derived from the previously published comprehensive review and high-resolution crystallographic data.

**Figure 3:**
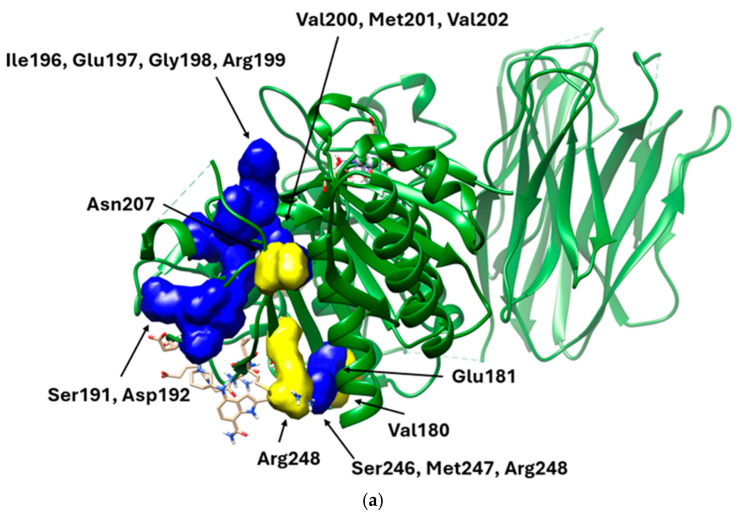

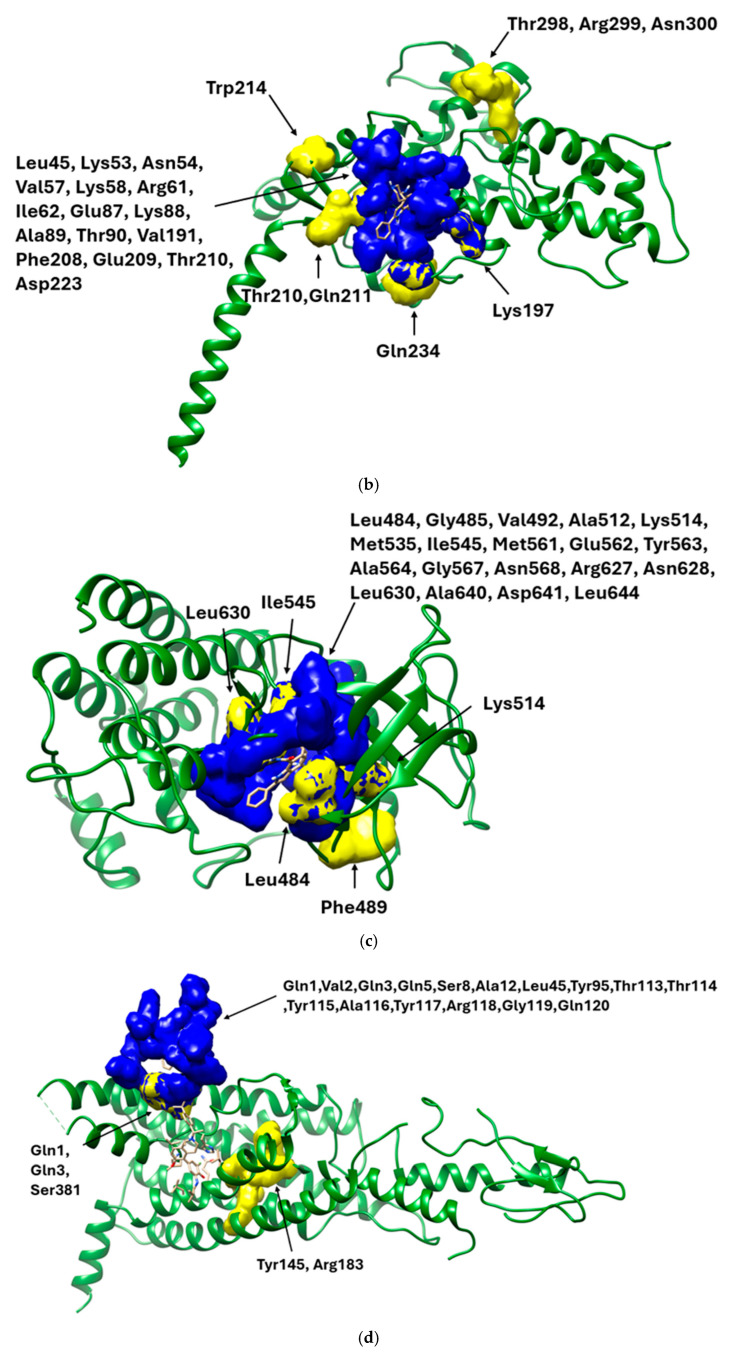

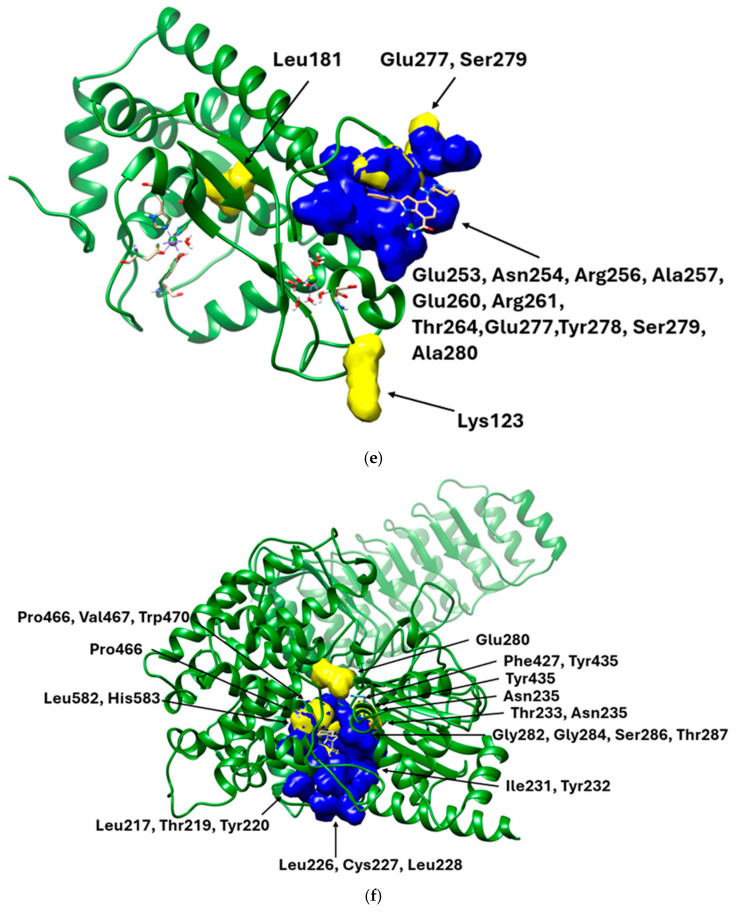
(**a**): Structural visualization of the human PCSK9 (PDB ID: 2P4E) docking environment using UCSF Chimera. The catalytic center (yellow surface) represents the functionally critical residues of the substrate-binding groove, whereas the predicted binding pocket of compound D4Z (blue surface) delineates the docking-derived site. (**b**): Structural visualization of the human GLP1R (PDB ID: 6X18) docking environment using UCSF Chimera. The orthosteric pocket (yellow surface) represents the functionally critical orthosteric residues within the transmembrane domain, as established in previous work, while the binding pocket of D4Z (blue surface) shows the docking-derived site. (**c**): Structural visualization of the human FGFR1 (PDB ID: 4RWI) docking environment using UCSF Chimera. The catalytic pocket (yellow surface) represents the ATP-binding residues within the kinase domain, while the binding pocket of D4Z (blue surface) depicts the docking-derived pose. (**d**): Structural visualization of the human GIPR (PDB ID: 7RBT) docking environment using UCSF Chimera. The orthosteric pocket (yellow surface) represents residues critical for incretin recognition, as established previously, whereas the binding pocket of D4Z (blue surface) corresponds to the docking-derived site. (**e**): Structural visualization of the human NF-κB p65 subunit (PDB ID: 4Q3J) docking environment using UCSF Chimera. The regulatory surface (yellow surface) represents functionally important residues identified as essential for transcriptional regulation, while the D4Z binding pocket (blue surface) reflects the docking-derived site. (**f**): Structural visualization of the human NLRP3 NACHT domain (PDB ID: 5IRM) docking environment using UCSF Chimera. The catalytic pocket (yellow surface) represents ADP-binding residues established previously, whereas the binding pocket of D4Z (blue surface) corresponds to the docking-derived pose.

The structural evaluation of the D4Z–PCSK9 complex indicates a high degree of correspondence between the binding site and the experimentally defined catalytic pocket. As reported previously, the catalytic domain is characterized by a specific topography involving residues Val180, Asn207, and Arg248, which are functionally critical for the regulation of lipid metabolism. Docking results for D4Z reveal a binding pocket (blue surface) that encompasses these catalytic coordinates (yellow surface), consistent with a docking score of −8.780 kcal/mol. The ligand orientation enables interactions with Glu181, Ser191, Asp192, Ile196, Glu197, Gly198, Arg199, Val200, Met201, Val202, Ser246, Met247, and Arg248 (Figure 3a). This extensive occupancy of the substrate-binding groove suggests that D4Z is computationally modeled to positionally overlap with the binding interface, suggesting a potential role as a steric shield. Notably, Arg248 is shared by both the experimentally defined catalytic center and the docking-derived binding pocket, further supporting the relevance of the predicted pose. The catalytic center was defined within the substrate-binding groove, a functionally critical surface for LDLR engagement, and the grid coordinates were centered on the crystallographically identified LDLR interface.

The structural investigation of the D4Z–GLP1R complex demonstrates alignment between the predicted binding site and the receptor orthosteric pocket. The functional domain of the human GLP-1 receptor is defined by a cluster of residues within the transmembrane helical bundle, including Lys197, Tyr205, Gln210, Gln211, Trp214, Gln234, Thr298, Arg299, and Asn300, which are involved in endogenous GLP-1 recognition and downstream signaling. Comparative visualization indicates that the D4Z binding pocket (blue surface) achieves near-complete occupancy of this catalytic region (yellow surface), with the ligand deeply sequestered within the orthosteric cavity and yielding a docking score of −10.280 kcal/mol. The observed complementarity is supported by interactions with Leu45, Lys53, Asn54, Val57, Lys58, Arg61, Ile62, Glu87, Lys88, Ala89, Thr90, Val191, Phe208, Glu209, Thr210, and Asp223 (Figure 3b). The identification of Thr210 as a shared residue between the orthosteric pocket and the D4Z binding site further supports the structural consistency of the model. The catalytic center was localized within the orthosteric pocket, deep in the transmembrane bundle, and the docking grid was generated from the coordinates of co-crystallized non-peptide agonists to ensure targeting of residues responsible for insulinotropic signaling.

The structural assessment of the D4Z–FGFR1 complex confirms that the ligand is positioned within the kinase ATP-binding cleft. According to previous work, this functional site is localized in the hinge region and adjacent areas that are essential for receptor autophosphorylation and downstream signaling, and is primarily defined by residues such as Leu484, Phe489, Lys514, Ile545, and Leu630. Visual analysis shows that the D4Z binding pocket (blue surface) overlaps with the orthosteric pocket (yellow surface), with the ligand embedded at the interface of the hinge region and activation loop and achieving a docking score of −10.432 kcal/mol. The stability of this orientation is supported by contacts with Leu484, Val492, Ala512, Lys514, Met535, Ile545, Met561, Glu562, Tyr563, Ala564, Gly567, Asn568, Arg627, Asn628, Leu630, Ala640, Asp641, and Leu644 (Figure 3c). The presence of Leu484, Lys514, and Ile545 as shared residues between the catalytic cleft and the D4Z binding site indicates that the ligand occupies the core functional framework of the kinase domain. The catalytic center was defined within the ATP-binding cleft, and the grid generation was guided by the position of the co-crystallized inhibitor in the 4RWI structure, with the aim of sterically hindering ATP entry and subsequent autophosphorylation events.

The structural analysis of the D4Z–GIPR complex indicates consistent targeting of the receptor’s primary activation domain. The orthosteric site of the human GIP receptor is characterized by residues within the transmembrane helices, including Gln1, Gln3, Tyr145, Arg183, and Ser381, which are essential for incretin binding and initiation of metabolic signaling. UCSF Chimera visualization shows that the D4Z binding pocket (blue surface) overlaps with this catalytic region (yellow surface), with the ligand deeply lodged within the orthosteric groove and displaying a docking score of −10.212 kcal/mol. This interaction is further stabilized by contacts with Gln1, Val2, Gln3, Gln5, Ser8, Ala12, Leu45, Tyr95, Thr113, Thr114, Tyr115, Ala116, Tyr117, Arg118, Gly119, and Gln120 (Figure 3d). The identification of Gln1 and Gln3 as shared residues between the catalytic pocket and the D4Z binding site supports the notion that the ligand is anchored at the primary activation gateway of the receptor. For GIPR, the catalytic center was defined as the orthosteric pocket at the interface of extracellular loops and the transmembrane core, and the docking results indicate that D4Z forms an interaction network spanning both the N and G chains, mimicking the binding footprint of the native GIP peptide.

The structural assessment of the D4Z–NF-κB complex reveals notable correspondence with the protein’s experimentally defined functional domains. The regulatory surface of the NF-κB p65 subunit includes residues such as Lys123, Leu181, Glu277, and Ser279, which are critical for transcriptional activity and DNA binding. Comparative visualization demonstrates that the D4Z binding pocket (blue surface) overlaps extensively with the regulatory surface (yellow surface), with the compound positioned within an interaction hub involving Glu253, Asn254, Arg256, Ala257, Glu260, Arg261, Thr264, Glu277, Tyr278, Ser279, and Ala280, and achieving a docking score of −8.115 kcal/mol (Figure 3e). The convergence of the blue and yellow surfaces, particularly around Glu277 and Ser279, suggests that D4Z may partially mask a functionally relevant portion of the regulatory surface. The catalytic site was centered on the DNA-binding interface of the p65 subunit, selected based on mutagenesis data implicating these residues in interactions with κB DNA promoter sequences, thereby providing a structural rationale for potential modulation of NF-κB–mediated transcription.

The structural evaluation of the D4Z–NLRP3 complex indicates convergence with the conserved nucleotide-binding domain of the NACHT region. The catalytic pocket is defined by residues including Asn235, Glu280, Tyr435, Pro466, and His583, which are essential for ADP/ATP binding and inflammasome oligomerization [4]. Visualization shows that the D4Z binding pocket (blue surface) occupies this catalytic region (yellow surface), with the ligand sequestered within the ADP-binding cleft and reaching the best docking score in the panel (−10.620 kcal/mol). The interaction is stabilized by contacts with Leu217, Thr219, Tyr220, Leu226, Cys227, Leu228, Ile231, Tyr232, Thr233, Asn235, Phe427, Tyr435, Pro466, Val467, Trp470, Gly282, Gly284, Ser286, Thr287, Leu582, and His583 (Figure 3f). The fact that Asn235, Tyr435, Pro466, and His583 are shared between the experimentally defined regulatory surface and the D4Z binding site supports the idea that the ligand occupies key nucleotide-binding coordinates involved in NLRP3 function. The catalytic pocket was defined based on the nucleotide-binding site within the NACHT domain by removing the native ADP from the 5IRM crystal structure and centering the grid on the vacated site; given that NLRP3 activation depends on ATP/ADP exchange, targeting this region suggests a potential mechanism by which D4Z might interfere with inflammasome assembly.

### 3.4. Desmond Molecular Dynamics Simulations of D4Z with the Six Targets

To evaluate the impact of D4Z on the local residue flexibility of the six therapeutic targets, Root-Mean-Square Fluctuation (RMSF) profiles were calculated for both the apo (ligand-free) and holo (D4Z-bound) states over a 200 ns trajectory. Across all targets, a consistent pattern was observed: the introduction of D4Z was associated with a reduction in fluctuation amplitudes within functionally critical regions, indicative of more conformationally constrained complexes.

#### 3.4.1. Stabilization of Metabolic and Kinase Targets (PCSK9 and FGFR1)

**Figure 4:**
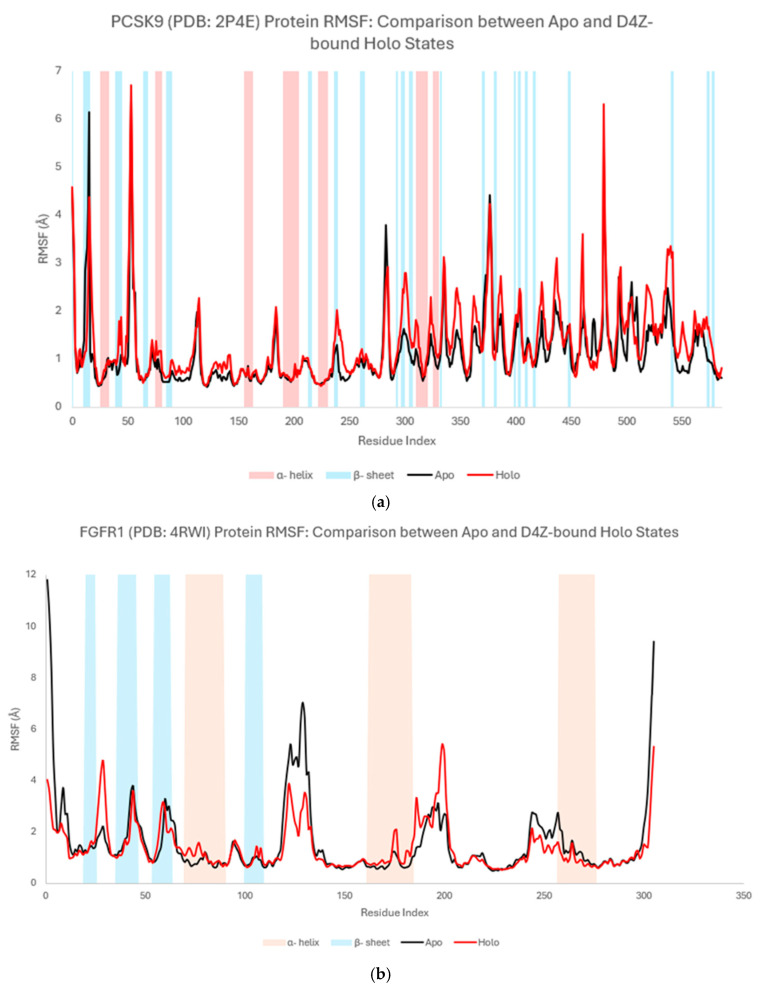

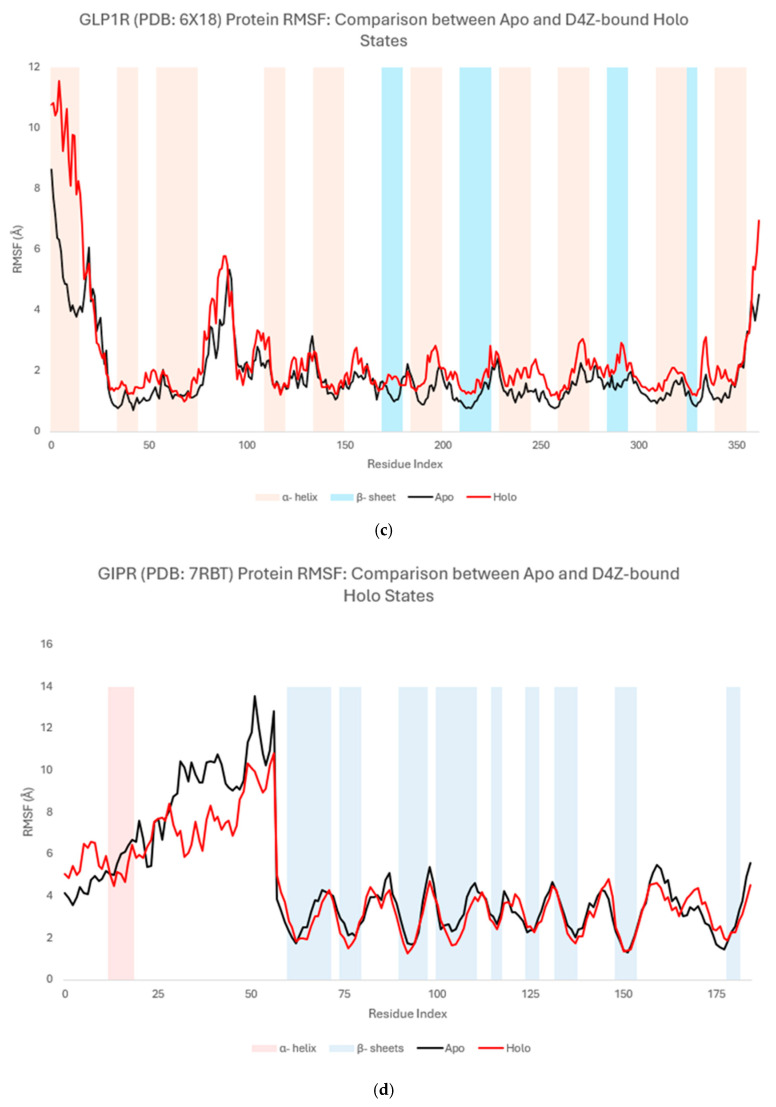

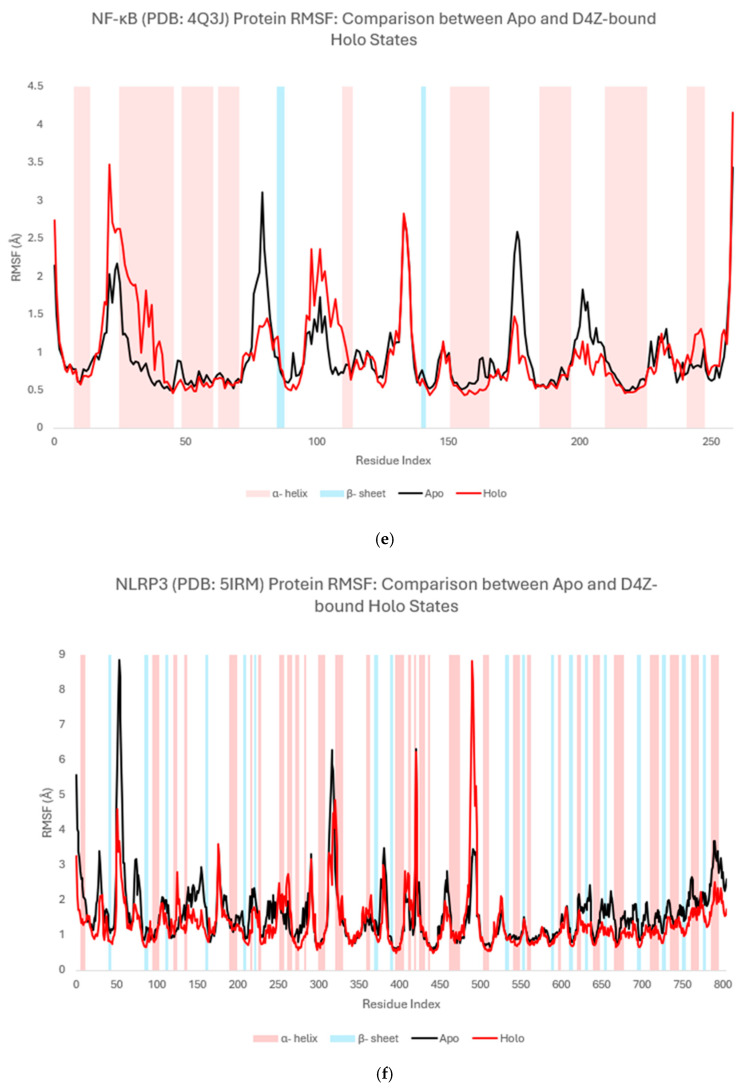
(**a**): Comparative RMSF profiles of PCSK9 (PDB ID: 2P4E) backbone atoms in the apo (black line) and D4Z-bound holo (red line) states during a 200 ns simulation at 310 K. The shaded background represents the secondary structural elements: α-helices (pink) and β-sheets (light blue). The attenuation of fluctuations in the holo state, particularly within the catalytic groove, illustrates the ligand-associated structural stabilization. (**b**): Comparative RMSF profiles of the FGFR1 kinase domain (PDB ID: 4RWI) in the apo (black line) and D4Z-bound holo (red line) states during a 200 ns simulation at 310 K. The shaded background represents secondary structural elements, specifically α-helices (pink) and β-sheets (light blue). The reduced fluctuations in the holo state, especially within the secondary structural motifs of the catalytic cleft, indicate ligand-induced stabilization of the kinase domain. (**c**): Comparative RMSF profiles of GLP1R (PDB ID: 6X18) in the apo (black line) and D4Z-bound holo (red line) states during a 200 ns simulation (310 K, POPC membrane). The shaded background colors represent secondary structural elements, specifically α-helices (pink) and β-sheets (light blue). The attenuation of fluctuations in the holo state, particularly within the transmembrane helices and orthosteric pocket regions, highlights D4Z-associated stabilization of the receptor. (**d**): Comparative RMSF profiles of GIPR (PDB ID: 7RBT) backbone atoms in the apo (black line) and D4Z-bound holo (red line) states. The simulation was conducted for 200 ns at 310 K under an NPγT ensemble within a POPC phospholipid bilayer. The shaded background identifies α-helices (pink) and β-sheets (light blue). The suppression of mobility in the holo state, particularly within the transmembrane helices and N-terminal region, indicates reduced conformational flexibility upon ligand binding. (**e**): Comparative RMSF profiles of the NF-κB p65 subunit (PDB ID: 4Q3J) backbone atoms in the apo (black line) and D4Z-bound holo (red line) states. The simulation was conducted for 200 ns at 310 K under an NPT ensemble. Shaded regions correspond to α-helices (pink) and β-sheets (light blue). The overall attenuation of fluctuations in the holo state reflects ligand-associated stabilization of the regulatory domain. (**f**): Comparative RMSF profiles of the NLRP3 NACHT domain (PDB ID: 5IRM) backbone atoms in the apo (black line) and D4Z-bound holo (red line) states. The simulation was conducted for 200 ns at 310 K under an NPT ensemble. Shaded background regions identify α-helices (pink) and β-sheets (light blue). The substantial reduction in fluctuations in the holo state, particularly within the ADP-binding pocket residues, indicates structural stabilization of the nucleotide-binding domain in the presence of D4Z.

For the catalytic domains of PCSK9 and FGFR1, D4Z behaves as a stabilizing structural element. In PCSK9 (Figure 4a), reduced flexibility was observed across the β-strands of the catalytic groove. Notable differences between the apo and holo states were detected in the residue ranges 150–250 and 280–350, where fluctuations exceeding 3–4 Å in the Apo state decreased predominantly to below 1.5 Å in the Holo state. Similarly, in the FGFR1 kinase domain (Figure 4b), D4Z reduced the mobility of the ATP-binding cleft. The most pronounced divergence occurred in the 110–140 residue range, where an Apo RMSF peak of approximately 7 Å was lowered to below 4 Å in the Holo form, suggesting a restriction of the conformational motions associated with catalytic activity.

#### 3.4.2. Anchoring of Transmembrane Incretin Receptors (GLP1R and GIPR)

Simulations in a POPC phospholipid bilayer suggested that D4Z contributes to the stabilization of GLP1R and GIPR by dampening the intrinsic flexibility of their transmembrane helical bundles. For GLP1R (Figure 4c), D4Z was associated with decreased fluctuations within the orthosteric pocket (residues 150–320), effectively smoothing RMSF peaks relative to the apo state. The effect was even more pronounced in GIPR (Figure 4d), where the intrinsically flexible N-terminal domain (residues 25–55) showed a reduction in peak RMSF values from approximately 14 Å in the apo simulation to below 10 Å in the holo state. This shift toward a more compact conformational ensemble suggests that D4Z can stabilize the receptor functional core in a dynamic membrane environment.

#### 3.4.3. Modulation of Inflammatory Gatekeepers (NF-κB and NLRP3)

For the inflammatory targets, D4Z was found to modulate the flexibility of regions linked to regulatory function. In the NF-κB p65 subunit (Figure 4e), reduced fluctuations were observed at the DNA-binding interface (residues 70–85), where RMSF values decreased from approximately 3.1 Å in the apo state to below 1.5 Å in the holo state. In the NLRP3 NACHT domain (Figure 4f), D4Z markedly attenuated mobility in the nucleotide-binding region, particularly in the 40–60 residue range, where an apo RMSF peak of around 9 Å was reduced to approximately 3.5 Å in the holo simulation. These changes are consistent with ligand-associated stabilization of conformations relevant to transcriptional and inflammasome-regulatory activity, respectively, while recognizing that functional inhibition must be experimentally confirmed.

#### 3.4.4. System Stability and Thermodynamic Equilibrium

To complement the local flexibility insights obtained from the RMSF profiles, backbone root-mean-square deviation (RMSD) time-series plots were presented for both the apo and holo states over the 200 ns trajectories (Figures are presented in Supplementary Materials Figures S5–S16).

For the soluble targets -comprising the enzyme PCSK9, the FGFR1 kinase, the NF-κB transcription factor and the NLRP3 inflammasome- the Cα protein backbones demonstrated robust structural convergence, establishing steady, well-behaved plateaus within a narrow 1.5–4.9 Å range during the second half of the simulations. Specifically, the D4Z-FGFR1 and D4Z-NLRP3 complexes exhibited structural consistency; the ligand locked into clefts within the first 15 ns, maintaining rigid configurations near 7.0 Å for FGFR1 and between 1.5 and 2.0 Å for NLRP3 until the end of the trajectories. Similarly, in the D4Z-PCSK9 complex, the small molecule achieved thermodynamic equilibrium during the final 25 ns (175–200 ns) with a stable binding pose near 2.0 Å. In complex D4Z-NF-κB the D4Z candidate established a structural lock at 142 ns, near 7–8 Å until the end of the trajectory. In comparison, apo forms of these soluble proteins converged smoothly, stabilizing at 3.0–3.5 Å for PCSK9, 4.4–4.9 Å for FGFR1, 1.8–2.1 Å for NF-κB, and 3.4–3.6 Å for NLRP3, which verifies that the underlying protein folds remained well-behaved.

For the membrane-embedded incretin receptors, GLP1R and GIPR, which were simulated within an explicit POPC phospholipid bilayer, the protein backbones exhibited higher structural deviation. This is fully consistent with the dynamic fluid properties of the lipid environment. Nevertheless, dynamic convergence for both GPCR systems was established, with the receptor backbones reaching stable equilibrium after the first 45–75 ns in the holo states, and after 100 ns in the unliganded apo forms. The D4Z candidate effectively navigated the flexible transmembrane orthosteric bundles between 4.5 and 5.5 Å for GLP1R, and stability between 13.0 and 16.5 Å for GIPR. Collectively, all investigated targets demonstrate that the 200 ns simulation window provided sufficient time to reach thermodynamic equilibrium.

#### 3.4.5. Ligand Structural Compactness and Contact Persistence Analyses

To evaluate ligand binding persistence and structural behavior beyond static representations, we performed the ligand radius of gyration (rGyr), molecular surface properties, and timeline interaction profiles (Further details are in Supplementary Materials Figures S17–S22). The stability of D4Z was corroborated by its highly steady radius of gyration (rGyr) profiles, which exhibited negligible fluctuations throughout the 200 ns course. This stability showcases that the ligand remains tightly bound and structurally anchored within its respective orthosteric or regulatory cavities, maintaining a stable, rigid-like bound conformation across diverse target environments. This structural consistency was further supported by highly uniform Molecular Surface Area (MolSA), Solvent Accessible Surface Area (SASA), and Polar Surface Area (PSA) values over the simulation timeline. Crucially, characterization of protein–ligand contact persistence and hydrogen-bond occupancy over the simulation course mapped an uninterrupted interaction network, demonstrating that the ligand establishes stability and high-frequency molecular contacts with key binding site residues. This dynamic persistence of specific hydrogen bonds, water-mediated bridges, and hydrophobic and π-π interactions provides dynamic justification for the structural stability of D4Z.

### 3.5. SwissADME Prediction of D4Z

Based on the computational assessment via the SwissADME platform, compound D4Z displays a favorable physicochemical profile in line with established criteria for orally active small molecules. It shows no violations of Lipinski’s “Rule of Five”, and also complies with the Ghose, Veber, Egan, and Muegge filters, supporting its predicted drug-like character. With a molecular weight of 334.41 g/mol, 4 rotatable bonds, and a topological polar surface area (TPSA) of 82.94 Å^2^, D4Z combines moderate molecular size with limited flexibility and balanced polarity. Its consensus log Po/wo of 2.58 and a bioavailability score of 0.55 are compatible with passive diffusion across biological membranes and acceptable predicted oral exposure.

In terms of pharmacokinetic behavior, D4Z is predicted to have high gastrointestinal (GI) absorption, suggesting suitability for systemic administration via the oral route. At the same time, it is classified as non-permeant to the blood–brain barrier (BBB), which may help limit central nervous system–related effects. Although identified as a substrate for P-glycoprotein (P-gp), the absence of PAINS or Brenk structural alerts, together with a synthetic accessibility score of 3.11, supports its feasibility as a lead compound from a medicinal chemistry perspective. The predicted inhibition of several cytochrome P450 isoforms (CYP1A2, CYP2C19, CYP2D6, and CYP3A4) offers an early indication of possible drug–drug interaction liabilities that will need to be explored experimentally in future studies (Table 2).

A comparative assessment of the lead candidates indicates that D4Z has a more favorable predicted pharmacokinetic and drug-likeness profile than compound 17X, which was not pursued further due to multiple liabilities. Although 17X showed attractive binding scores, SwissADME analysis revealed two major Lipinski violations related to its higher molecular weight (>500 Da) and lipophilicity (MLOGP > 4.15), along with several failures in the Ghose, Egan, and Muegge filters. These features are reflected in a low bioavailability score of 0.17, suggesting limited oral absorption compared with the 0.55 score of D4Z. In addition, its pronounced hydrophobicity and classification as insoluble raise concerns for systemic administration, while a synthetic accessibility score of 5.14 points to increased complexity for chemical production. In contrast, D4Z retains full compliance with the evaluated bioavailability criteria and a more favorable synthetic accessibility (3.11), supporting its selection as the preferred lead for further cardiometabolic-oriented optimization.

### 3.6. pkCSM Prediction of D4Z

The pharmacokinetic evaluation of compound D4Z using the pkCSM server suggests a generally favorable predicted absorption and distribution profile. The molecule is estimated to display high human intestinal absorption (90.76%) and moderate Caco-2 permeability (0.817 log P), which is consistent with efficient uptake following oral administration. With a predicted steady-state volume of distribution (VDss) of 1.076 log L/kg, D4Z is expected to distribute between plasma and peripheral tissues to a moderate extent. Although the compound is predicted to act as a P-glycoprotein substrate, its blood–brain barrier (BBB) permeability (−0.606 log BB) and CNS permeability (−2.268 log PS) fall within ranges compatible with limited central nervous system penetration, which may reduce the likelihood of CNS-related adverse effects. The metabolic profile further indicates that D4Z is a CYP3A4 substrate and a CYP1A2 inhibitor, highlighting potential metabolic clearance routes and points of attention for possible drug–drug interactions.

In terms of predicted safety and excretion, D4Z shows a total clearance of 1.069 log mL/min/kg, indicative of a moderate elimination rate. The in silico toxicity assessment suggests an overall acceptable safety window: the compound is predicted to be negative for AMES mutagenicity and skin sensitization. At the same time, pkCSM flags a potential risk of hERG II channel inhibition and hepatotoxicity, which will require careful experimental evaluation. The predicted maximum tolerated dose (0.286 log mg/kg/day), L50 (2.597 mol/kg), and rat LOAEL (1.567 log mg/kg_bw/day) are consistent with a toxicity profile that may be manageable during lead optimization, provided that liver function and cardiac repolarization are specifically monitored in subsequent in vivo studies (Table 3).

### 3.7. Proposed Synthetic Route of D4Z

Following the in silico evaluation of compound D4Z as a multitargeting agent, a feasible proposed synthetic strategy was outlined to enable its chemical preparation for future in vitro and in vivo biological studies. The proposed route was designed with the aid of a retrosynthetic search in the Reaxys medicinal chemistry database, prioritizing commercially available starting materials, high atom economy, and scalable reaction conditions.

**Figure 5:**
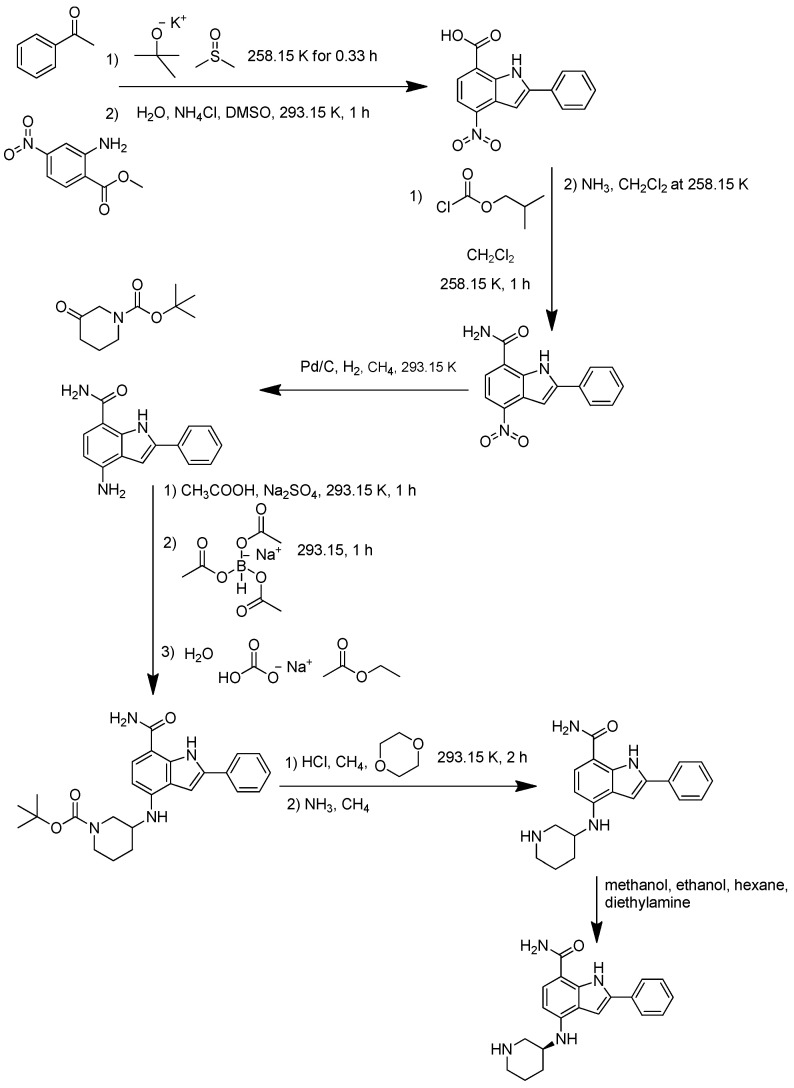
Proposed synthetic route for compound D4Z as generated and refined using the Reaxys database. The scheme summarizes the key chemical transformations, reagents, and reaction conditions required for the assembly of D4Z from commercially available precursors.

As illustrated in Figure 5, the total proposed synthesis of D4Z relies on a convergent, multi-step sequence centered on the construction of the indole core and subsequent introduction of the piperidine and amide moieties. The proposed pathway is consistent with a synthetic accessibility (SA) score of 3.11, in agreement with the SwissADME prediction that D4Z should be accessible by standard organic synthesis, in addition to its predicted pharmacological properties.

## 4. Discussion

The paradigm shift in modern pharmacology from the classical “one-target, one-drug” model to multitarget drug design reflects the need to address complex, network-driven pathologies such as metabolic syndrome. In this study, D4Z was investigated as a novel small-molecule scaffold rationally designed to engage multiple nodes within the interconnected axes of lipid dysregulation and chronic low-grade inflammation. Across docking and molecular dynamics analyses, D4Z displayed a balanced thermodynamic profile, with docking scores (−8.115 to −10.620 kcal/mol) against six distinct therapeutic targets, suggesting that simultaneous modulation of metabolic and inflammatory pathways may be achievable within a single chemotype.

A key outcome of the structural analysis was the close correspondence between the predicted D4Z binding pockets and experimentally defined catalytic or orthosteric sites for all six proteins. The substantial overlap between the docking-derived surfaces and the known functional regions provides a mechanistic rationale for the proposed multitarget activity. This is particularly apparent for the NLRP3 NACHT domain, where D4Z was predicted to occupy the conserved ATP/ADP-binding pocket and displace the native nucleotide [4]. In this model, the ligand establishes an extended network of hydrogen bonds, π–π interactions, and hydrophobic contacts with residues essential for nucleotide recognition, which is consistent with a potential stabilization of conformations less permissive to inflammasome activation.

The interaction pattern observed for PCSK9 further illustrates how D4Z may intervene at critical regulatory interfaces. The docking and pocket-overlap analyses indicate that D4Z occupies the substrate-binding groove and interacts with a cluster of residues that includes the shared Arg248 [14], a residue previously implicated in LDL receptor recognition. Such positioning suggests that D4Z could function as a steric and chemical shield at the PCSK9–LDLR interface and thereby support LDLR preservation, conceptually aligning with the therapeutic goal of PCSK9 inhibition while retaining the practical advantages of a small molecule over monoclonal antibodies, particularly in terms of oral delivery and manufacturing complexity.

Furthermore, this dual targeting scheme directly addresses the complex network of upstream metabolic and endocrine mediators that govern vascular remodeling. Recent evidence highlights how systemic metabolic axes and homeostatic proteins, such as the GH-IGF-1 axis and S-Klotho, converge on atherosclerosis to modulate endothelial integrity, macrophage polarization and vascular muscle cell viability. By inhibiting PCSK9-mediated lipid accumulation and engaging the FGFR1 kinase domain, D4Z aligns with this modern cardiometabolic rationale. This multitarget design offers the potential to suppress vascular inflammation, reinforce fibrous cap stability and modify vulnerable lesions to a healed phenotype [24].

In the context of glycemic control, D4Z demonstrated high docking affinity and structurally coherent binding poses within the orthosteric pockets of GLP1R and GIPR. For GLP1R, the ligand engages residues within the transmembrane helical bundle, including Thr210, which is shared between the orthosteric pocket definition and the docking-derived site, supporting the model that D4Z addresses a functionally relevant region of the receptor. In the GIPR complex, D4Z forms contacts across both the N and G chain [25] and interacts with residues Gln1 and Gln3 identified as part of the orthosteric domain. Together with the observed reduction in RMSF values within the transmembrane helices and N-terminal domain in the holo simulations, these findings suggest that D4Z can stabilize conformations compatible with incretin receptor modulation in a membrane environment.

The compound’s predicted engagement of the FGFR1 kinase domain and NF-κB p65 subunit extends its potential impact beyond lipid and incretin signaling to broader cardiometabolic and inflammatory pathways [12]. Within FGFR1, D4Z occupies the ATP-binding cleft and interacts with hinge and activation-loop residues such as Leu484, Lys514, and Ile545, suggesting a mode of binding reminiscent of ATP-competitive kinase inhibitors and consistent with modulation of FGF-driven metabolic signaling. For NF-κB, the binding pocket of D4Z overlaps with a regulatory surface enriched in residues implicated in DNA binding, including Glu277 and Ser279, and MD simulations indicate reduced flexibility in this region upon ligand binding. These observations support a model in which D4Z may hinder protein–DNA or protein–cofactor interactions required for full transcriptional activation, although this remains to be validated experimentally.

The 200 ns MD simulations provided dynamic support for the docking-based hypotheses by showing that D4Z is associated with reduced backbone fluctuations in regions that coincide with catalytic or regulatory motifs. For soluble targets such as PCSK9, FGFR1, NF-κB, and NLRP3, RMSF analyses revealed attenuation of motion within the substrate-binding groove, ATP-binding cleft, DNA-binding interface, and nucleotide-binding pocket, respectively. For the membrane-embedded incretin receptors, holo simulations in a POPC bilayer showed decreased mobility within the transmembrane helices and orthosteric cavities, together with reduced disorder in the flexible GIPR N-terminus. Although RMSF changes cannot be equated directly with functional inhibition, the consistent pattern of locally decreased flexibility supports the view that D4Z stabilizes well-defined binding modes across structurally diverse targets. Crucially, because the lipid bilayer environment inherently dampens transmembrane domain fluctuations compared to aqueous solutions, these RMSF profiles were interpreted within each individual target apo/holo framework rather than through direct cross-system magnitude comparisons.

Beyond its immediate vasculoprotective effects, decreasing low-grade cardiometabolic inflammation carries profound prognostic relevance regarding downstream myocardial protection. Chronic systemic inflammation serves as a critical pathobiologic driver of cardiac structural remodeling, instigating pathological cascades (myocardial hypertrophy, interstitial fibrosis and the progression to chronic heart failure). The ability of D4Z to target and suppress central inflammatory nodes like the NLRP3 inflammasome and the NF-κB p65 subunit extends its therapeutic utility and conventional metabolic endpoints [26].

From a translational standpoint, the combined SwissADME and pkCSM analyses suggest that D4Z possesses an encouraging in silico ADMET profile. The compound complies with multiple drug-likeness filters (Lipinski, Ghose, Veber, Egan, Muegge), exhibits predicted high GI absorption, and shows a bioavailability score compatible with oral administration. It is also predicted to have limited BBB and CNS permeability, which may reduce central nervous system involvement, and to be synthetically accessible with moderate complexity (synthetic accessibility score 3.11). At the same time, pkCSM flags D4Z as a substrate for CYP3A4 and an inhibitor of CYP1A2, and predicts potential hERG II inhibition and hepatotoxicity, underscoring the need for focused experimental assessment of cardiac and hepatic safety, as well as drug–drug interaction risks. When compared to the alternative candidate 17X, which shows multiple rule violations, low predicted oral bioavailability, and poorer solubility and synthetic accessibility, D4Z emerges as the more promising lead structure for further optimization.

Given that cardiometabolic patients are typically difficult to manage co-existing clusters of metabolic and cardiovascular disorders, the predicted liabilities of hERG II inhibition and hepatotoxicity carry clinical significance to avoid adverse drug–drug interactions. To address these off-target profiles, a structured lead optimization strategy should be deployed in developmental phases. Specifically, computational strategies such as Matched Molecular Pair Analysis (MMPA) can be utilized to investigate how they affect the molecule’s toxicological profile. Furthermore, fine-tuning the scaffold through bioisosteric replacement of structural motifs linked to hERG channel blockage such as altering core basic centers to optimize the overall lipophilicity. A clear medicinal chemistry path to retain multitarget affinity while minimizing cardiac and hepatic safety risks.

From a methodological standpoint, combining a SwissSimilarity-based analog search with structure-based docking, molecular dynamics and in silico ADMET profiling proved advantageous. The ligand-based virtual screening step ensured that D4Z, 17X and K4F were selected from experimentally validated bioactive chemical space, while the subsequent docking and 200 ns MD simulations highlighted D4Z as the compound with the most balanced, high-affinity and dynamically stable multitarget profile across PCSK9, GLP1R, FGFR1, GIPR, NF-κB and NLRP3. In parallel, SwissADME and pkCSM predictions indicated that D4Z combines an overall drug-like physicochemical and pharmacokinetic profile with manageable liabilities, thereby supporting its designation as a computationally prioritized lead structure that warrants experimental synthesis and validation.

### Limitations

Several limitations of this study should be acknowledged. First, all findings are based on in silico methodologies, including docking, molecular dynamics, and ADMET/toxicity prediction platforms, and therefore remain subject to the inherent approximations and biases of these computational models. No experimental binding, functional, or pharmacokinetic data are currently available for D4Z, and the predicted multitarget activity as well as the ADMET and toxicity profiles must be verified in vitro and in vivo before any firm conclusions can be drawn. Second, the docking protocol assumes relatively rigid receptor conformations and may not fully capture induced fit or long-timescale conformational rearrangements, despite the inclusion of 200 ns MD simulations. Third, ADMET and toxicity models such as SwissADME and pkCSM, while widely used, are associated with non-negligible rates of false positives and false negatives, particularly for hERG, hepatotoxicity, and CYP inhibition predictions, and should be considered as hypothesis-generating rather than definitive. Finally, although the selected six targets form a mechanistically coherent cardiometabolic–inflammatory axis, they do not encompass the full complexity of metabolic syndrome pathophysiology, and off-target effects, system-level network responses, and inter-individual variability were not addressed in this work.

Finally, a challenge in prioritizing multitarget chemotypes is the potential risk of off-target activity and cross-reactivity across the human proteome. Because D4Z was designed to accommodate structurally diverse binding pockets, the present computational screening cannot definitively rule out binding toward close structural homologues. Specifically, the lack of a comprehensive selectivity panel or counter-screening assays against alternative FGFR isoforms (such as FGFR2–4) or other kinases sharing similar ATP-binding folds represents a limitation that remains to be addressed. Conducting structured in silico profiling and in vitro selectivity screens against structurally related target families must be considered for the upcoming experimental follow-up and lead optimization phases to ensure a therapeutic safety window.

Taken together, the present computational data support D4Z as a multitarget small-molecule lead with structurally rational engagement of key cardiometabolic and inflammatory nodes and a broadly favorable, yet still hypothetical, pharmacokinetic and toxicity profile. These results should be regarded as a mechanistically informed in silico proof-of-concept that motivates synthesis, biological evaluation and further optimization, and they illustrate how rational multitarget design can be leveraged to explore integrated therapeutic strategies for the cardiometabolic–inflammatory axis.

## 5. Conclusions

The present study outlines the rational design and multi-level in silico evaluation of D4Z, a novel small molecule conceived to target multiple components of the cardiometabolic–inflammatory axis. By evolving the initial lead scaffold 1a into a multitarget candidate, D4Z was predicted to occupy functionally important catalytic and orthosteric pockets in PCSK9, GLP1R, FGFR1, GIPR, NF-κB, and NLRP3, with strong docking scores. The observed correspondence between docking-derived binding sites and experimentally defined functional domains provides a structural basis for the proposed modulation of lipid metabolism, incretin signaling, and inflammatory pathways.

Molecular dynamics simulations over 200 ns further supported the stability of these complexes, indicating reduced backbone flexibility in regions that coincide with catalytic or regulatory motifs in all six targets. The systematic attenuation of RMSF values in holo trajectories, particularly within substrate-binding grooves, ATP-binding clefts, orthosteric pockets, DNA-binding interfaces, and nucleotide-binding sites, is consistent with ligand-associated stabilization of conformational states that may be less compatible with full target activation. This effect was especially evident for NLRP3 and NF-κB, where reduced mobility in nucleotide- and DNA-binding regions suggests a potential for modulating key drivers of chronic inflammatory signaling, pending experimental confirmation.

Complementary SwissADME and pkCSM analyses indicate that D4Z combines predicted drug-like physicochemical properties with high gastrointestinal absorption, moderate distribution, limited blood–brain barrier penetration, and acceptable synthetic accessibility. At the same time, in silico toxicity and metabolism models highlight potential risks, including CYP inhibition, hERG II liability, and hepatotoxicity, which will need to be addressed through targeted in vitro and in vivo studies. When compared to alternative scaffolds such as 17X, which exhibits multiple rule violations, low predicted bioavailability, and higher synthetic complexity, D4Z emerges as a more balanced lead structure for further optimization.

Overall, these computational findings support D4Z as a promising multitarget small-molecule lead that merits chemical synthesis and experimental evaluation as a potential modulator of interconnected cardiometabolic and inflammatory pathways. More broadly, the work illustrates how integrated docking, molecular dynamics, ADMET prediction, and retrosynthetic analysis can be combined to generate mechanistically informed hypotheses and prioritize candidates for next-step validation in the development of multitarget therapies for metabolic syndrome.

## Figures and Tables

**Table 1:** Βinding affinities (kcal/mol) of selected compounds (D4Z, 17X, K4F, 1a and reference ligands danuglipron, parthenolide, erdafitinib, MK-0893, SBC-115076, MCC-950) against six therapeutic targets. Molecular docking simulations were performed on the following PDB structures: PCSK9 (2P4E), GLP1R (6X18), FGFR1 (4RWI), GIPR (7RBT), NFκB (4Q3J), and NLRP3 (5IRM). Lower (more negative) values indicate a stronger binding score and higher complex stability. Chemical structures for each compound are provided in the respective column.

Compound	Chemical Structure	PCSK9 (PDB ID: 2P4E)	GLP1R (PDB ID: 6X18)	FGFR1 (PDB ID: 4RWI)	GIPR (PDB ID: 7RBT)	NFκΒ (PDB ID: 4Q3J)	NLRP3 (PDB ID: 5IRM)
D4Z	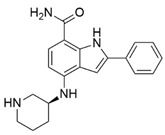	−8.780	−10.280	−10.432	−10.212	−8.115	−10.620
17X	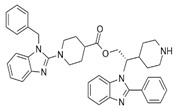	−5.594	−9.541	−10.320	−11.320	−7.780	−12.270
K4F	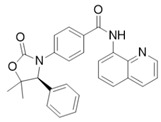	−3.018	−6.916	−8.371	−4.925	−3.016	−6.046
1a	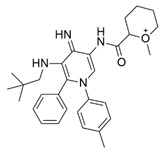	−3.215	−2.416	−1.100	−2.923	−2.480	−2.500
Danuglipron	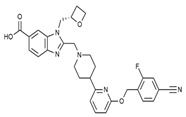	-	−9.504	-	-	-	-
Parthenolide	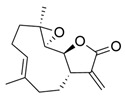	-	-	-	-	−7.102	-
Erdafitinib	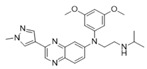	-	-	−10.401	-	-	-
MK-0893	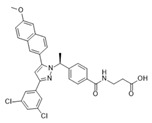	-	-	-	−9.770	-	-
SBC-115076	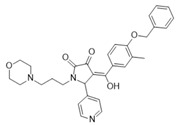	−7.265	-	-	-	-	-
MCC-950	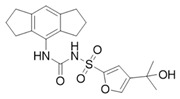	-	-	-	-	-	−9.430

**Table 2:** Parameters calculated for D4Z using the SwissADME web server.

Property	Descriptor	Value/Result
Physicochemical	Formula	C_20_H_22_N_4_O
	Molecular weight	334.41 g/mol
	Num. rotatable bonds	4
	TPSA	82.94 Å^2^
Lipophilicity	Consensus Log Po/w	2.58
Solubility	Log S (ESOL)	−3.79 (Soluble)
Pharmacokinetics	GI absorption	High
	BBB permeant	No
	P-gp substrate	Yes
Drug-likeness	Lipinski Violations	0
	Bioavailability Score	0.55
Medicinal Chemistry	PAINS/Brenk Alerts	0/0
	Synthetic Accessibility	3.11

**Table 3:** Predicted pharmacokinetic and toxicological properties of compound D4Z calculated using the pkCSM platform.

Category	Model Name	Predicted Value	Unit/Result
Absorption	Intestinal absorption (human)	90.762	% Absorbed
	Caco2 permeability	0.817	log P 10^−6^ (cm/s)
Distribution	VDss (human)	1.076	log L/kg
	BBB permeability	−0.606	log BB
	CNS permeability	−2.268	log PS
Metabolism	CYP3A4 substrate	Yes	Categorical
	CYP1A2 inhibitor	Yes	Categorical
Excretion	Total Clearance	1.069	log mL/min/kg
Toxicity	AMES toxicity	No	Negative
	hERG II inhibitor	Yes	Positive
	Oral Rat Acute Toxicity (LD50)	2.597	mol/kg
	Hepatotoxicity	Yes	Positive

## Data Availability

The original contributions presented in this study are included in the article. Further inquiries can be directed to the corresponding author.

## Supplementary Materials

The following supporting information can be downloaded at: https://www.mdpi.com/article/10.3390/biomedicines14061213/s1, Figures S1–S4: SwissADME prediction of compounds, Figures S5–S16: RMSD, Figures S17–S22: Molecular Dynamics Ligand Properties Tranjectory Analysis.

## Abbreviations

The following abbreviations are used in this manuscript:

GLP1R	Glucagon Peptide 1 Receptor
FGFR1	Fibroblast Growth Factor Receptor 1
GIPR	Glucose-Dependent Insulinotropic Polypeptide Receptor
PCSK9	Proprotein Convertase Subtilisin/Kexin Type 9
NLRP3	Nucleotide Binding Oligomerization Domain Like Receptor Protein 3
NF-κB	Nuclear Factor NF-Kappa-B p65 Subunit
