# Supplementary material for: Targeting the Crosstalk Between Metabolism and Chronic Inflammation: In Silico Multitargeting Drug Design Approach for Cardiometabolic Syndrome
Source: Biomedicines. 2026 May 27;14(6):1213. doi: 10.3390/biomedicines14061213 (PMC13296646; doi:10.3390/biomedicines14061213)
Supplement: Supplementary file 1 [file biomedicines-14-01213-s001.zip › biomedicines-4326258-supplementary.pdf]

Supplementary Materials

Targeting the Crosstalk between Metabolism and Chronic Inflammation: *In silico* Multitargeting Drug Design Approach for Metabolic Syndrome

Errikos Petsas[1], Gerasimos Siasos[2], Thomas Mavromoustakos[1], Christos T. Chasapis[1]*

[1] *Laboratory of Organic Chemistry, Department of Chemistry, National and Kapodistrian University of Athens, Athens, Greece; errpets@chem.uoa.gr (E.P.), tmavrom@chem.uoa.gr (T.M.), cchasapis@chem.uoa.gr (C.T.C.)*

[2] *3rd Department of Cardiology, Thoracic Diseases General Hospital Sotiria, Medical School, National and Kapodistrian University of Athens, 11527 Athens, Greece; gsiasos@med.uoa.gr (G.S.)*

* *Correspondence: cchasapis@chem.uoa.gr (C.T.C.)*

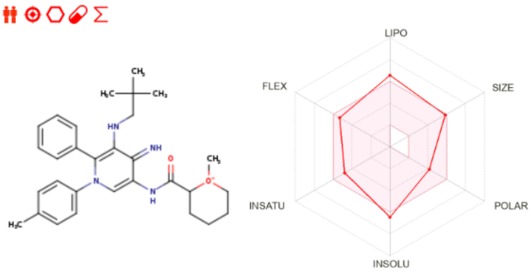

SMILES C[O+]1CCCCC1C(=O)Nc1cn(c2ccc(cc2)C)c(c(c1=N)NCC(C)(C)C)c1ccccc1

Physicochemical Properties	
Formula	C30H39N4O2+
Molecular weight	487.66 g/mol
Num. heavy atoms	36
Num. arom. heavy atoms	18
Fraction Csp3	0.40
Num. rotatable bonds	8
Num. H-bond acceptors	3
Num. H-bond donors	3
Molar Refractivity	149.35
TPSA	79.14 Å²

Lipophilicity	
Log $P_{o/w}$ (iLOGP)	1.01
Log $P_{o/w}$ (XLOGP3)	5.96
Log $P_{o/w}$ (WLOGP)	5.68
Log $P_{o/w}$ (MLOGP)	2.99
Log $P_{o/w}$ (SILICOS-IT)	4.82
Consensus Log $P_{o/w}$	4.09

Water Solubility	
Log *S* (ESOL)	-6.46
Solubility	1.69e-04 mg/ml ; 3.47e-07 mol/l
Class	Poorly soluble
Log *S* (Ali)	-7.40
Solubility	1.95e-05 mg/ml ; 4.00e-08 mol/l
Class	Poorly soluble
Log *S* (SILICOS-IT)	-9.78
Solubility	8.13e-08 mg/ml ; 1.67e-10 mol/l
Class	Poorly soluble

Pharmacokinetics	
GI absorption	High
BBB permeant	No
P-gp substrate	Yes
CYP1A2 inhibitor	No
CYP2C19 inhibitor	Yes
CYP2C9 inhibitor	No
CYP2D6 inhibitor	Yes
CYP3A4 inhibitor	Yes
Log K_p (skin permeation)	-5.04 cm/s

Druglikeness	
Lipinski	Yes; 0 violation
Ghose	No; 4 violations: MW>480, WLOGP>5.6, MR>130, #atoms>70
Veber	Yes
Egan	Yes
Muegge	No; 1 violation: XLOGP3>5
Bioavailability Score	0.55

Medicinal Chemistry	
PAINS	0 alert
Brenk	1 alert: charged_oxygen_sulfur
Leadlikeness	No; 3 violations: MW>350, Rotors>7, XLOGP3>3.5
Synthetic accessibility	4.97

Figure S1: SwissADME prediction of 1a.

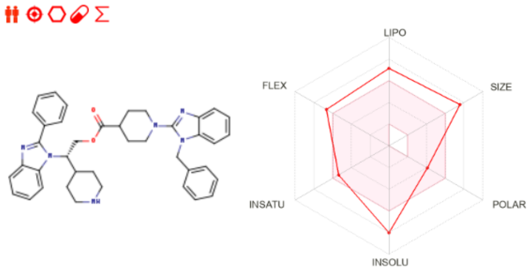

SMILES O=C(C1CCN(CC1)c1nc2c(n1Cc1ccccc1)cccc2)OC[C@@H](n1c(nc2c1cccc2)c1ccccc1)C1CCNCC1

Physicochemical Properties	
Formula	C40H42N6O2
Molecular weight	638.80 g/mol
Num. heavy atoms	48
Num. arom. heavy atoms	30
Fraction Csp3	0.33
Num. rotatable bonds	10
Num. H-bond acceptors	5
Num. H-bond donors	1
Molar Refractivity	198.78
TPSA	77.21 Å²

Lipophilicity	
Log $P_{o/w}$ (iLOGP)	4.54
Log $P_{o/w}$ (XLOGP3)	7.00
Log $P_{o/w}$ (WLOGP)	6.34
Log $P_{o/w}$ (MLOGP)	5.22
Log $P_{o/w}$ (SILICOS-IT)	5.53
Consensus Log $P_{o/w}$	5.73

Water Solubility	
Log *S* (ESOL)	-8.01
Solubility	6.20e-06 mg/ml ; 9.70e-09 mol/l
Class	Poorly soluble
Log *S* (Ali)	-8.44
Solubility	2.34e-06 mg/ml ; 3.66e-09 mol/l
Class	Poorly soluble
Log *S* (SILICOS-IT)	-11.22
Solubility	3.86e-09 mg/ml ; 6.04e-12 mol/l
Class	Insoluble

Pharmacokinetics	
GI absorption	High
BBB permeant	No
P-gp substrate	No
CYP1A2 inhibitor	No
CYP2C19 inhibitor	No
CYP2C9 inhibitor	No
CYP2D6 inhibitor	Yes
CYP3A4 inhibitor	Yes
Log K_p (skin permeation)	-5.23 cm/s

Druglikeness	
Lipinski	No; 2 violations: MW>500, MLOGP>4.15
Ghose	No; 4 violations: MW>480, WLOGP>5.6, MR>130, #atoms>70
Veber	Yes
Egan	No; 1 violation: WLOGP>5.88
Muegge	No; 3 violations: MW>600, XLOGP3>5, #rings>7
Bioavailability Score	0.17

Medicinal Chemistry	
PAINS	0 alert
Brenk	0 alert
Leadlikeness	No; 3 violations: MW>350, Rotors>7, XLOGP3>3.5
Synthetic accessibility	5.14

Figure S2: SwissADME prediction of 17X.

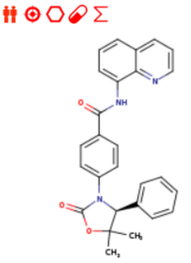

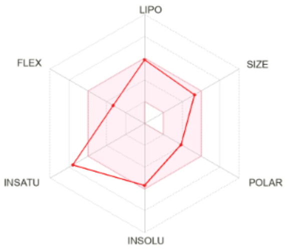

SMILES O=C(c1ccc(cc1)N1C(=O)OC([C@@H]1c1ccccc1)(C)C)Nc1cccc2c1nccc2

Physicochemical Properties	
Formula	C27H23N3O3
Molecular weight	437.49 g/mol
Num. heavy atoms	33
Num. arom. heavy atoms	22
Fraction Csp3	0.15
Num. rotatable bonds	5
Num. H-bond acceptors	4
Num. H-bond donors	1
Molar Refractivity	131.61
TPSA	71.53 Å²

Lipophilicity	
Log $P_{o/w}$ (iLOGP)	3.39
Log $P_{o/w}$ (XLOGP3)	4.71
Log $P_{o/w}$ (WLOGP)	5.07
Log $P_{o/w}$ (MLOGP)	3.72
Log $P_{o/w}$ (SILICOS-IT)	4.18
Consensus Log $P_{o/w}$	4.21

Water Solubility	
Log *S* (ESOL)	-5.68
Solubility	9.08e-04 mg/ml ; 2.07e-06 mol/l
Class	Moderately soluble
Log *S* (Ali)	-5.94
Solubility	5.01e-04 mg/ml ; 1.15e-06 mol/l
Class	Moderately soluble
Log *S* (SILICOS-IT)	-8.95
Solubility	4.90e-07 mg/ml ; 1.12e-09 mol/l
Class	Poorly soluble

Pharmacokinetics	
GI absorption	High
BBB permeant	No
P-gp substrate	Yes
CYP1A2 inhibitor	No
CYP2C19 inhibitor	Yes
CYP2C9 inhibitor	Yes
CYP2D6 inhibitor	No
CYP3A4 inhibitor	Yes
Log K_p (skin permeation)	-5.62 cm/s

Druglikeness	
Lipinski	Yes; 0 violation
Ghose	No; 1 violation: MR>130
Veber	Yes
Egan	Yes
Muegge	Yes
Bioavailability Score	0.55

Medicinal Chemistry	
PAINS	0 alert
Brenk	0 alert
Leadlikeness	No; 2 violations: MW>350, XLOGP3>3.5
Synthetic accessibility	3.66

Figure S3: SwissADME prediction of K4F.

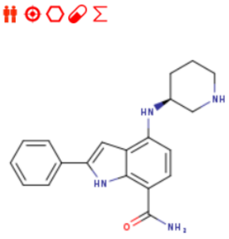

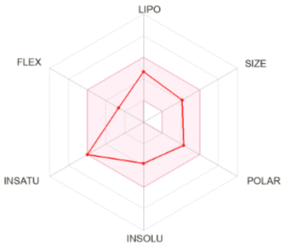

SMILES NC(=O)c1ccc(c2c1[nH]c(c2)c1ccccc1)N[C@H]1CCCNC1

Physicochemical Properties	
Formula	C20H22N4O
Molecular weight	334.41 g/mol
Num. heavy atoms	25
Num. arom. heavy atoms	15
Fraction Csp3	0.25
Num. rotatable bonds	4
Num. H-bond acceptors	2
Num. H-bond donors	4
Molar Refractivity	104.97
TPSA	82.94 Å²

Lipophilicity	
Log $P_{o/w}$ (iLOGP)	2.62
Log $P_{o/w}$ (XLOGP3)	2.70
Log $P_{o/w}$ (WLOGP)	2.53
Log $P_{o/w}$ (MLOGP)	1.88
Log $P_{o/w}$ (SILICOS-IT)	3.15
Consensus Log $P_{o/w}$	2.58

Water Solubility	
Log *S* (ESOL)	-3.79
Solubility	5.37e-02 mg/ml ; 1.61e-04 mol/l
Class	Soluble
Log *S* (Ali)	-4.09
Solubility	2.69e-02 mg/ml ; 8.04e-05 mol/l
Class	Moderately soluble
Log *S* (SILICOS-IT)	-6.62
Solubility	7.99e-05 mg/ml ; 2.39e-07 mol/l
Class	Poorly soluble

Pharmacokinetics	
GI absorption	High
BBB permeant	No
P-gp substrate	Yes
CYP1A2 inhibitor	Yes
CYP2C19 inhibitor	Yes
CYP2C9 inhibitor	No
CYP2D6 inhibitor	Yes
CYP3A4 inhibitor	Yes
Log K_p (skin permeation)	-6.42 cm/s

Druglikeness	
Lipinski	Yes; 0 violation
Ghose	Yes
Veber	Yes
Egan	Yes
Muegge	Yes
Bioavailability Score	0.55

Medicinal Chemistry	
PAINS	0 alert
Brenk	0 alert
Leadlikeness	Yes
Synthetic accessibility	3.11

Figure S4: SwissADME prediction of D4Z.

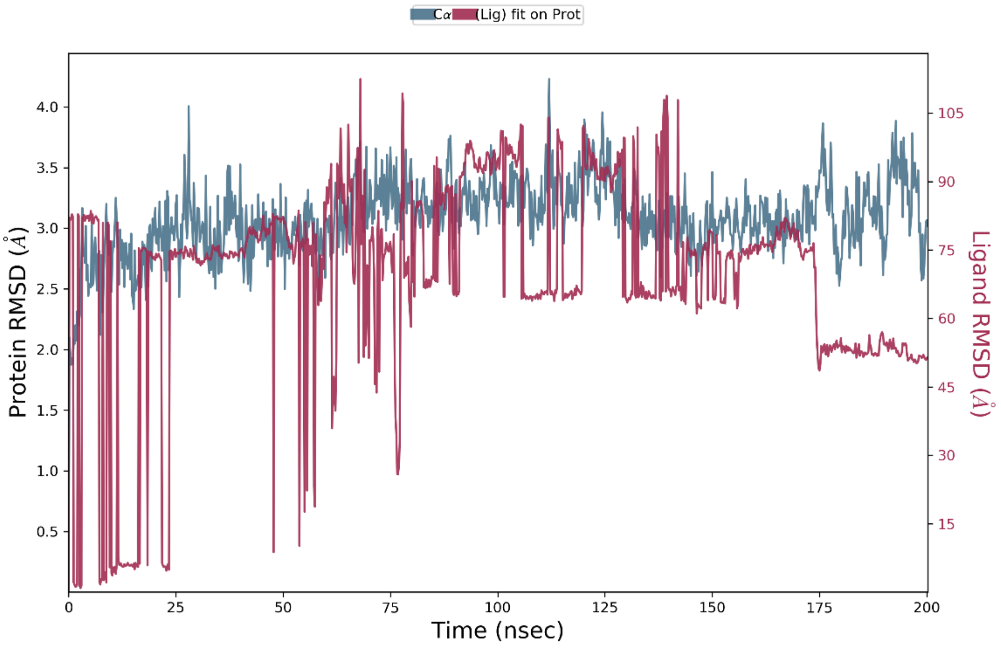

Figure S5: Root-mean-square deviation (RMSD) time-series plot for the Cα atoms of the human PCSK9 protein (PDB ID: 2P4E) (blue line, left axis) and the bound D4Z candidate (red line, right axis) over the 200 ns molecular dynamics trajectory.

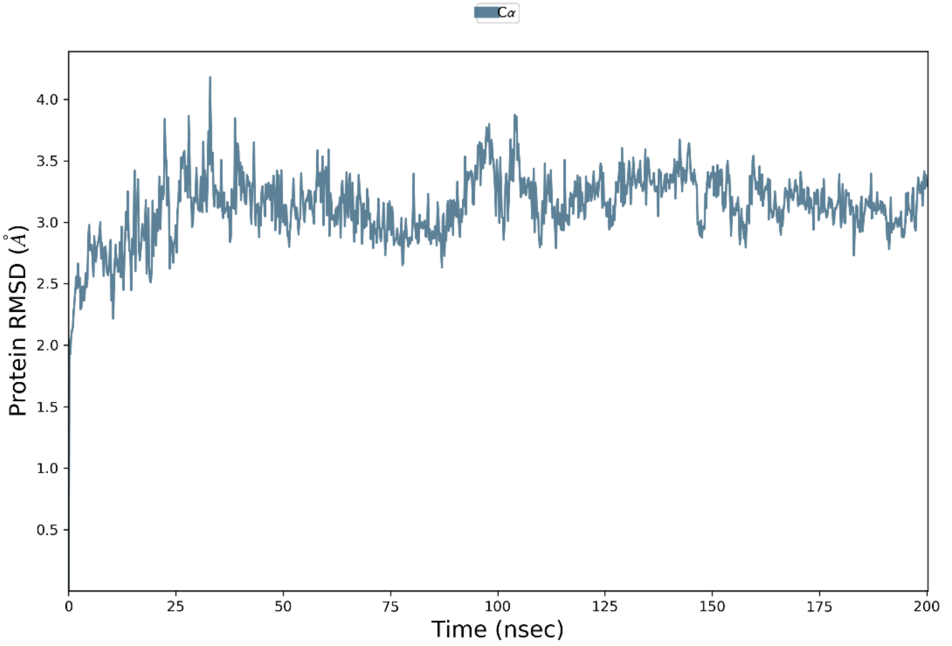

Figure S6: Root-mean-square deviation (RMSD) time-series plot for the Cα atoms of the human PCSK9 (PDB ID: 2P4E) protein during the 200 ns molecular dynamics simulation.

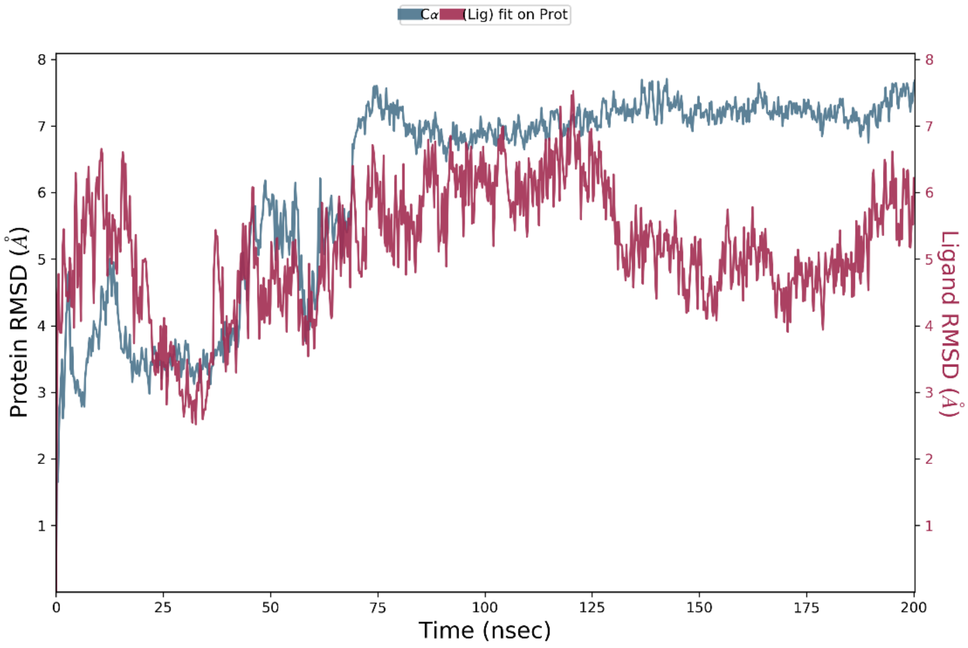

Figure S7: Root-mean-square deviation (RMSD) time-series plot for the Cα atoms of the membrane-embedded GLP1R receptor in a POPC bilayer (blue line, left axis) and the bound D4Z candidate (red line, right axis) over the 200 ns trajectory.

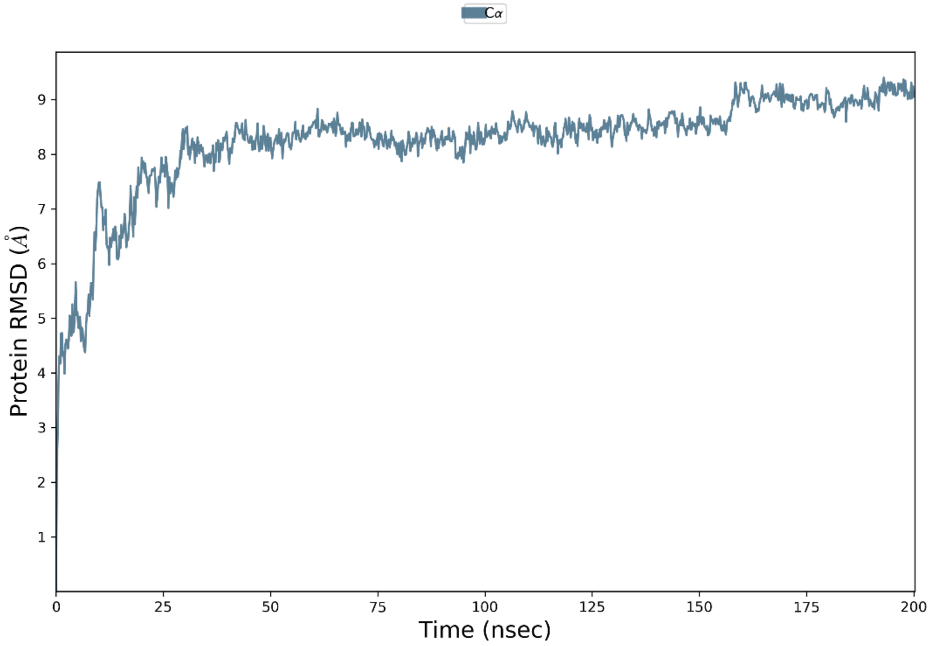

Figure S8: Root-mean-square deviation (RMSD) time-series plot for the Cα atoms of the human GLP1R receptor in its apo state during the 200 ns molecular dynamics simulation within a POPC phospholipid bilayer.

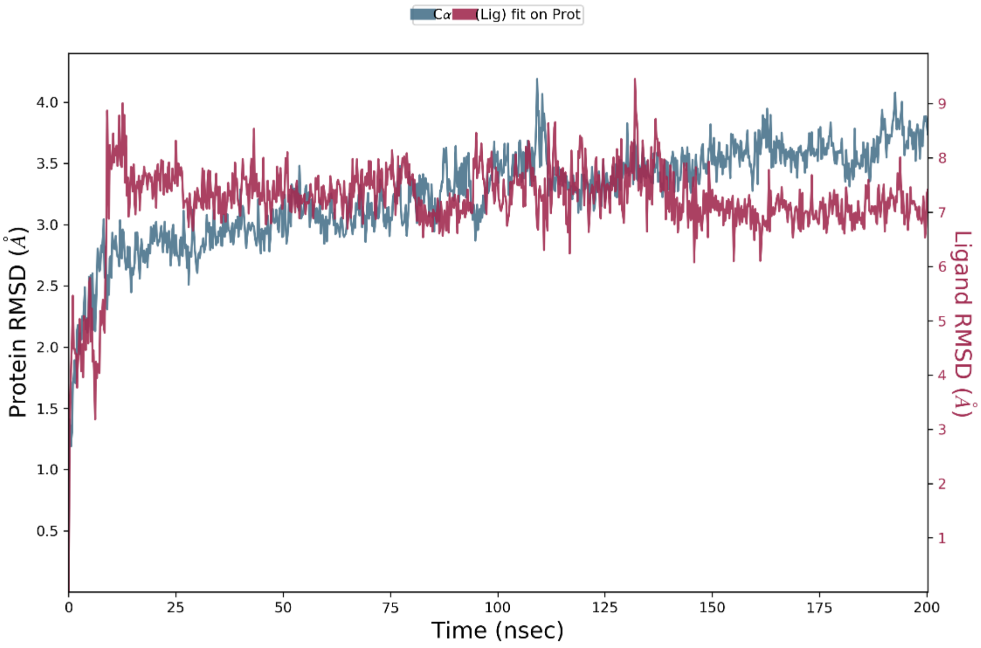

Figure S9: Root-mean-square deviation (RMSD) time-series plot for the Cα atoms of the FGFR1 (PDB ID: 4RWI) kinase domain (blue line, left axis) and the bound D4Z candidate (red line, right axis) over the 200 ns molecular dynamics simulation in an explicit water box.

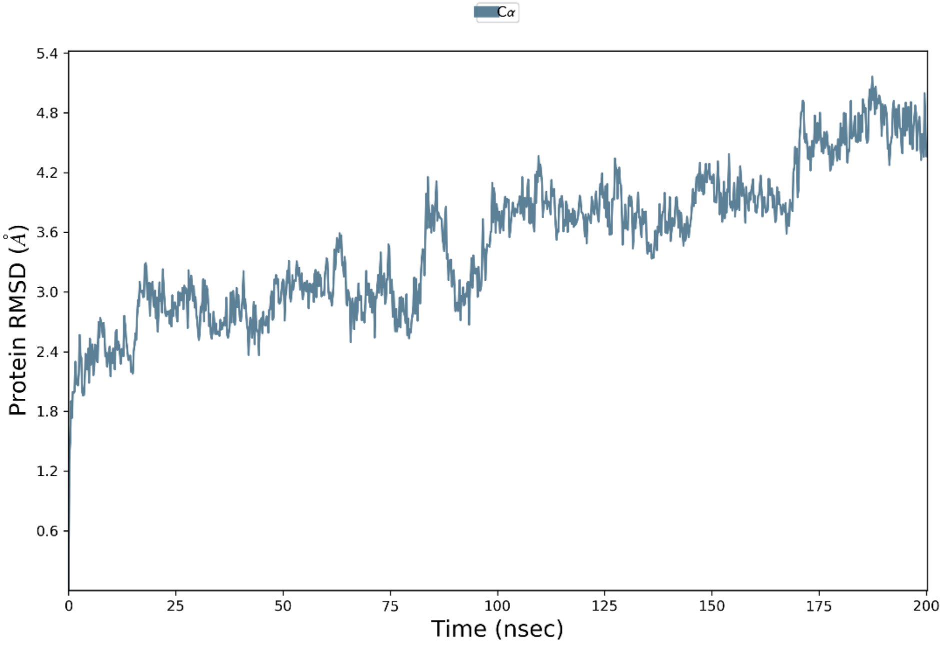

Figure S10: Root-mean-square deviation (RMSD) time-series plot for the Cα atoms of the FGFR1 (PDB ID: 4RWI) kinase domain during the 200 ns molecular dynamics simulation.

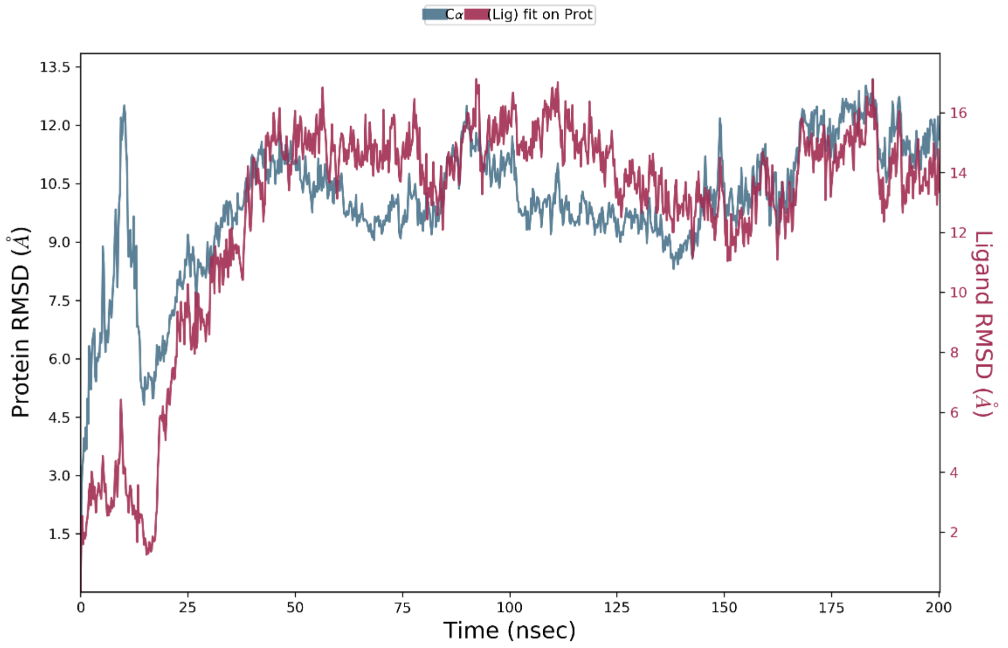

Figure S11: Root-mean-square deviation (RMSD) time-series plot for the Cα atoms of the membrane-embedded GIP receptor (PDB ID: 7RBT) in a POPC bilayer (blue line, left axis) and the bound D4Z candidate (red line, right axis) over the 200 ns trajectory.

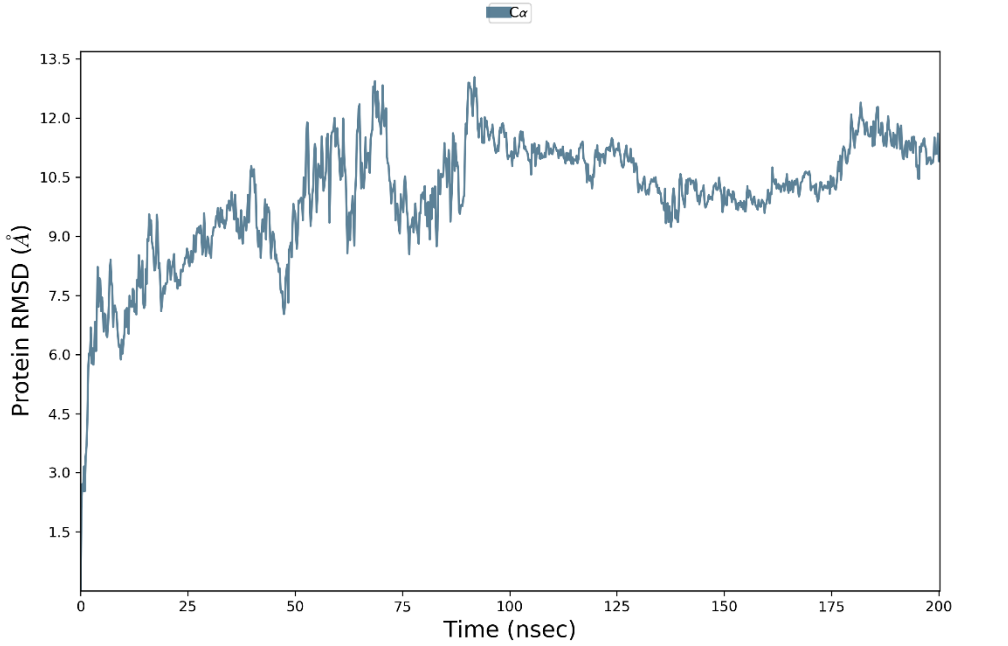

Figure S12: Root-mean-square deviation (RMSD) time-series plot for the Cα atoms of the human GIPR receptor (PDB ID: 7RBT) during the 200 ns molecular dynamics simulation within a POPC phospholipid bilayer.

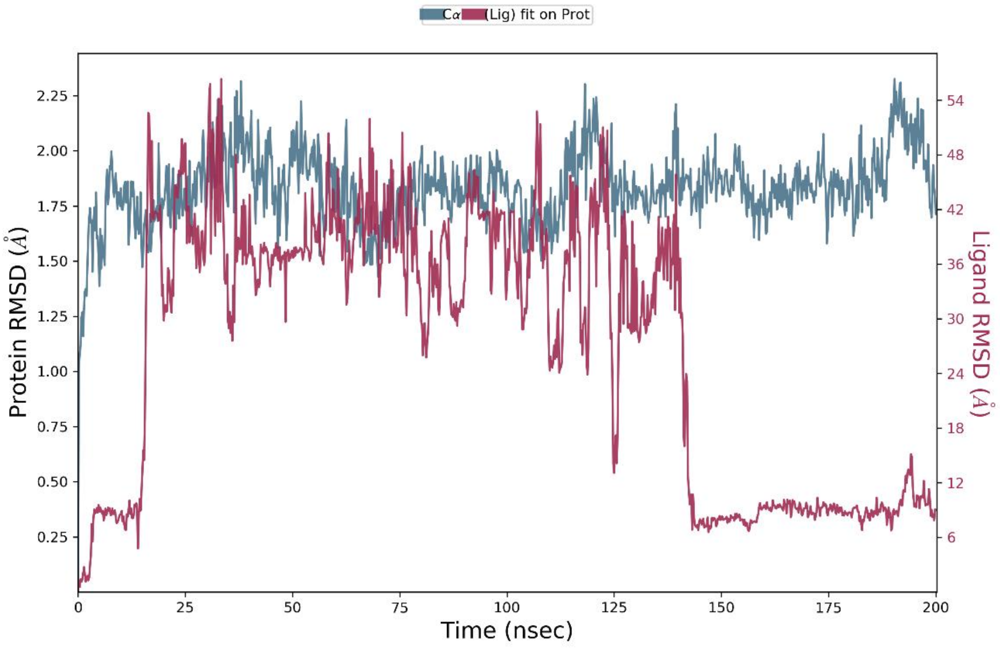

Figure S13: Root-mean-square deviation (RMSD) time-series plot for the Cα atoms of the human NF-κB p65 subunit (PDB ID: 4Q3J) (blue line, left axis) and the bound D4Z candidate (red line, right axis) over the 200 ns molecular dynamics trajectory in an explicit water box.

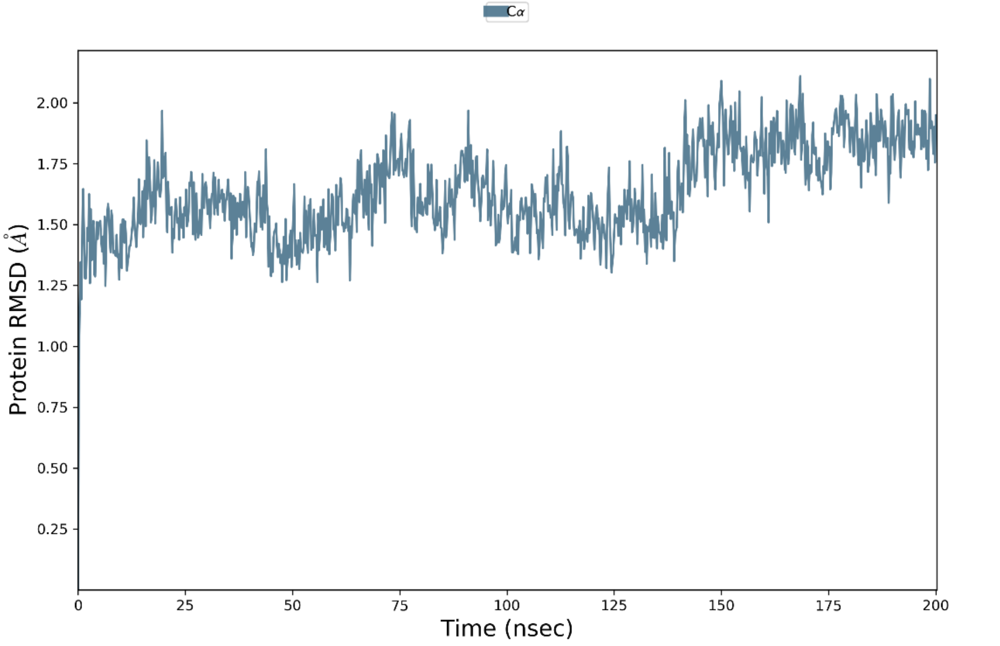

Figure S14: Root-mean-square deviation (RMSD) time-series plot for the Cα atoms of the human NF-κB p65 subunit (PDB ID: 4Q3J) during the 200 ns molecular dynamics simulation.

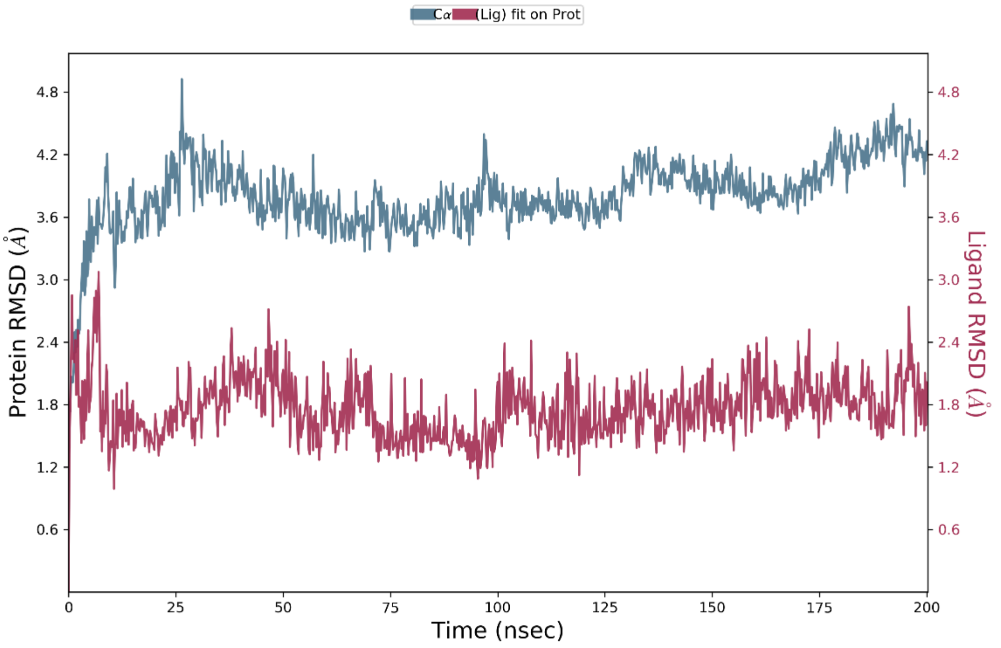

Figure S15: Root-mean-square deviation (RMSD) time-series plot for the Cα atoms of the human NLRP3 inflammasome (PDB ID: 5IRM) (blue line, left axis) and the bound D4Z candidate (red line, right axis) over the 200 ns molecular dynamics trajectory in an explicit water box.

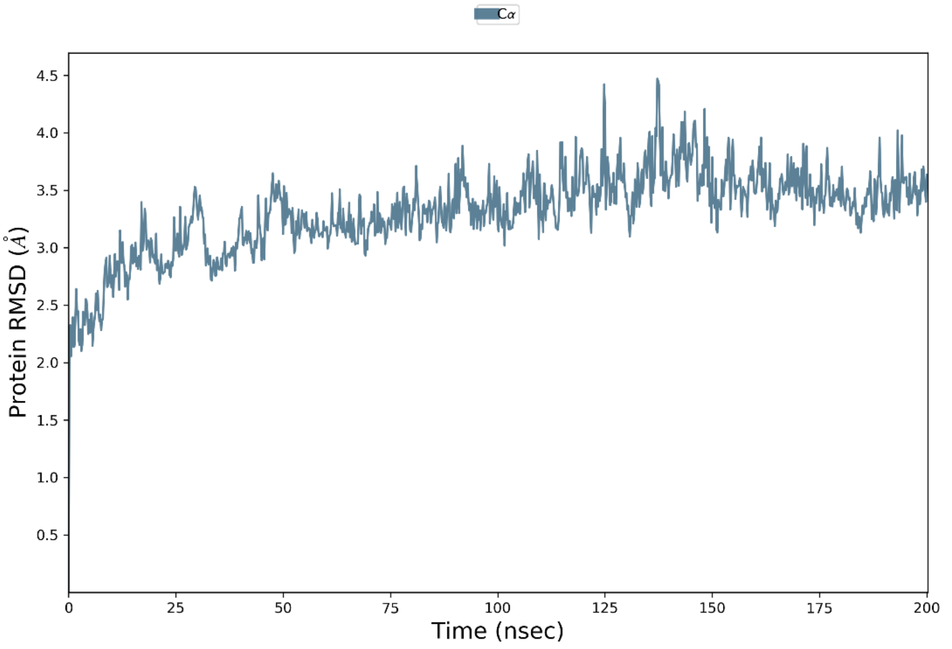

Figure S16: Root-mean-square deviation (RMSD) time-series plot for the Cα atoms of the human NLRP3 inflammasome (PDB ID: 5IRM) during the 200 ns molecular dynamics simulation.

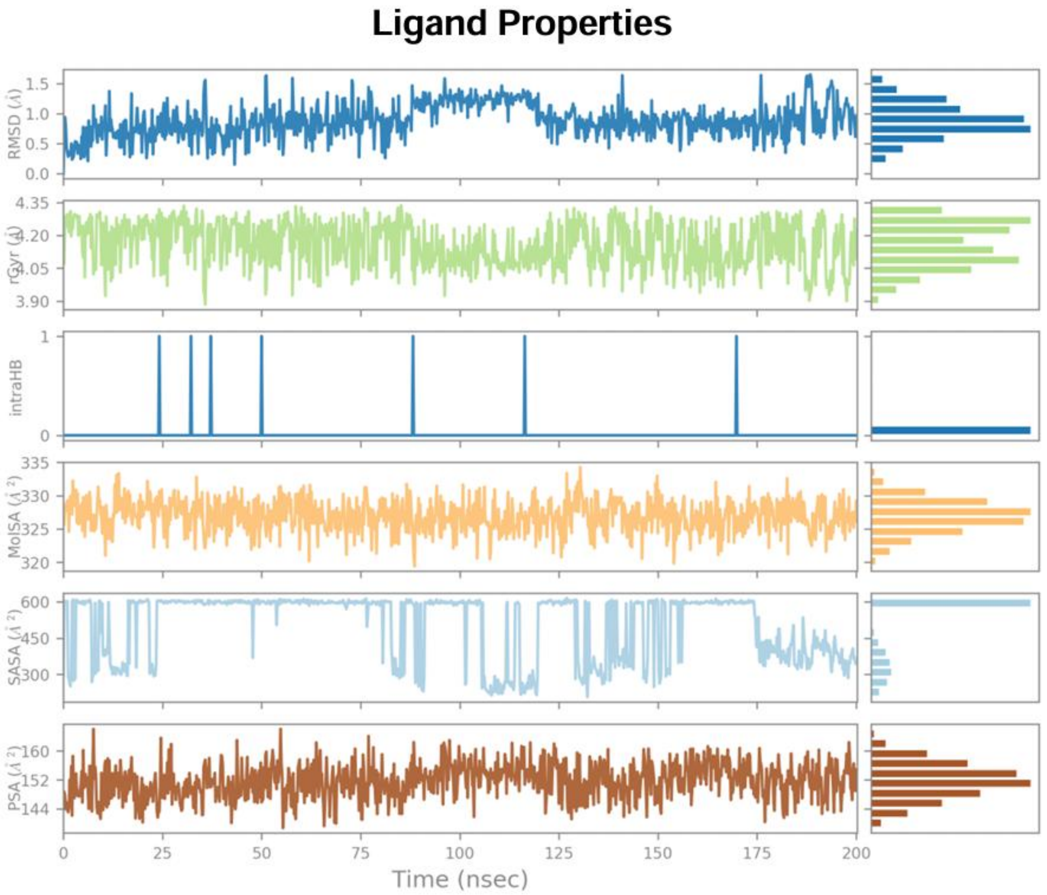

Figure S17: Molecular dynamics ligand properties trajectory analysis of PCSK9 (PDB ID: 2P4E) in complex with D4Z over 200 ns. (Top to Bottom) Ligand RMSD monitoring confirming system equilibration and stable binding; ligand properties (rGyr, intraHB, MolSA, SASA, PSA) confirming conformational compactness; and protein–ligand contact histograms with timeline persistence mapping the predicted high-occupancy interaction network.

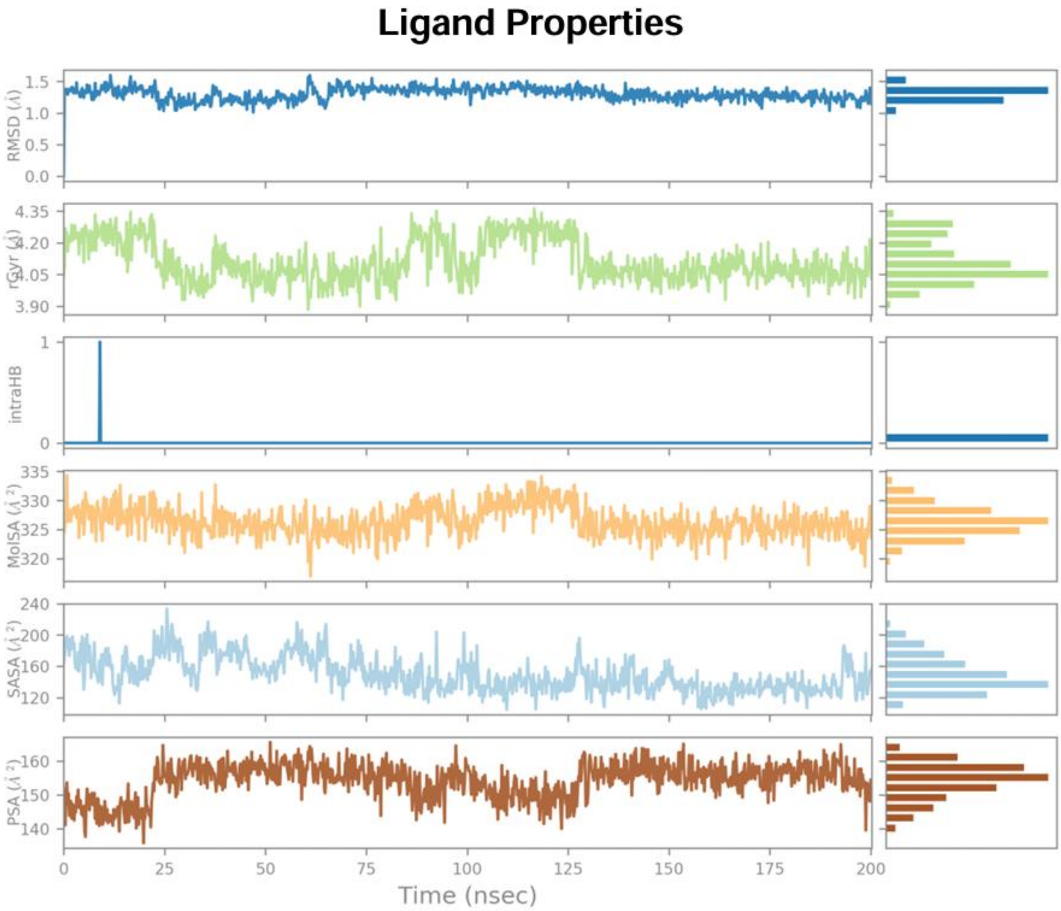

Figure S18: Molecular dynamics ligand properties trajectory analysis of GLP1R (PDB ID: 6X18) in complex with D4Z over 200 ns. (Top to Bottom) Ligand RMSD monitoring confirming system equilibration and stable binding; ligand properties (rGyr, intraHB, MolSA, SASA, PSA) confirming conformational compactness; and protein–ligand contact histograms with timeline persistence mapping the predicted high-occupancy interaction network.

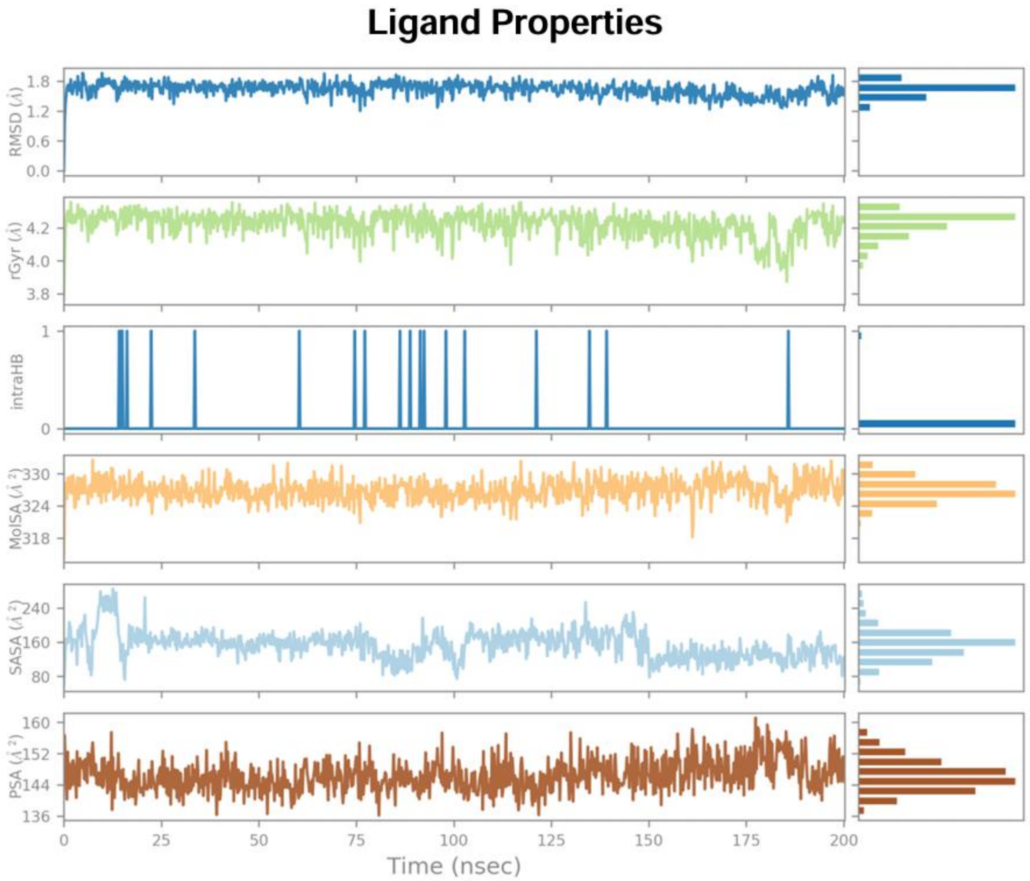

Figure S19: Molecular dynamics ligand properties trajectory analysis of FGFR1 (PDB ID: 4RWI) in complex with D4Z over 200 ns. (Top to Bottom) Ligand RMSD monitoring confirming system equilibration and stable binding; ligand properties (rGyr, intraHB, MolSA, SASA, PSA) confirming conformational compactness; and protein–ligand contact histograms with timeline persistence mapping the predicted high-occupancy interaction network.

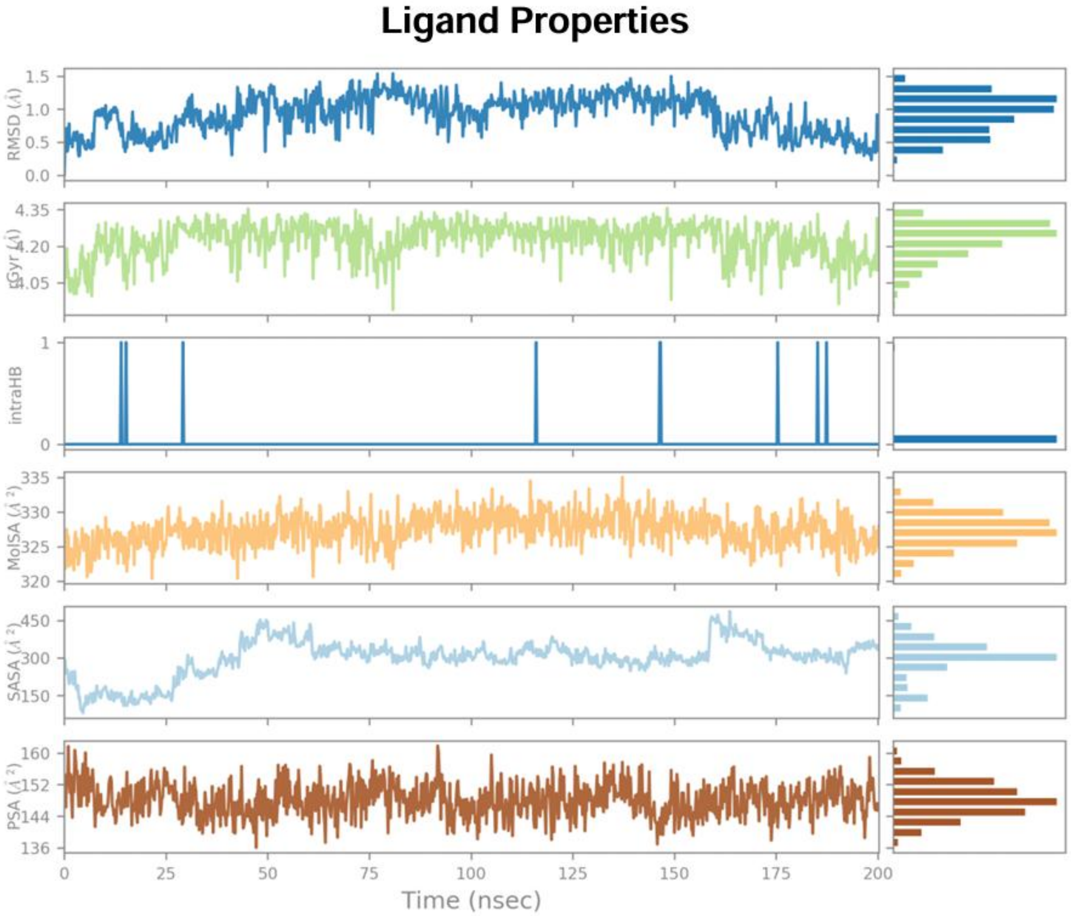

Figure S20: Molecular dynamics ligand properties trajectory analysis of GIPR (PDB ID: 7RBT) in complex with D4Z over 200 ns. (Top to Bottom) Ligand RMSD monitoring confirming system equilibration and stable binding; ligand properties (rGyr, intraHB, MolSA, SASA, PSA) confirming conformational compactness; and protein–ligand contact histograms with timeline persistence mapping the predicted high-occupancy interaction network.

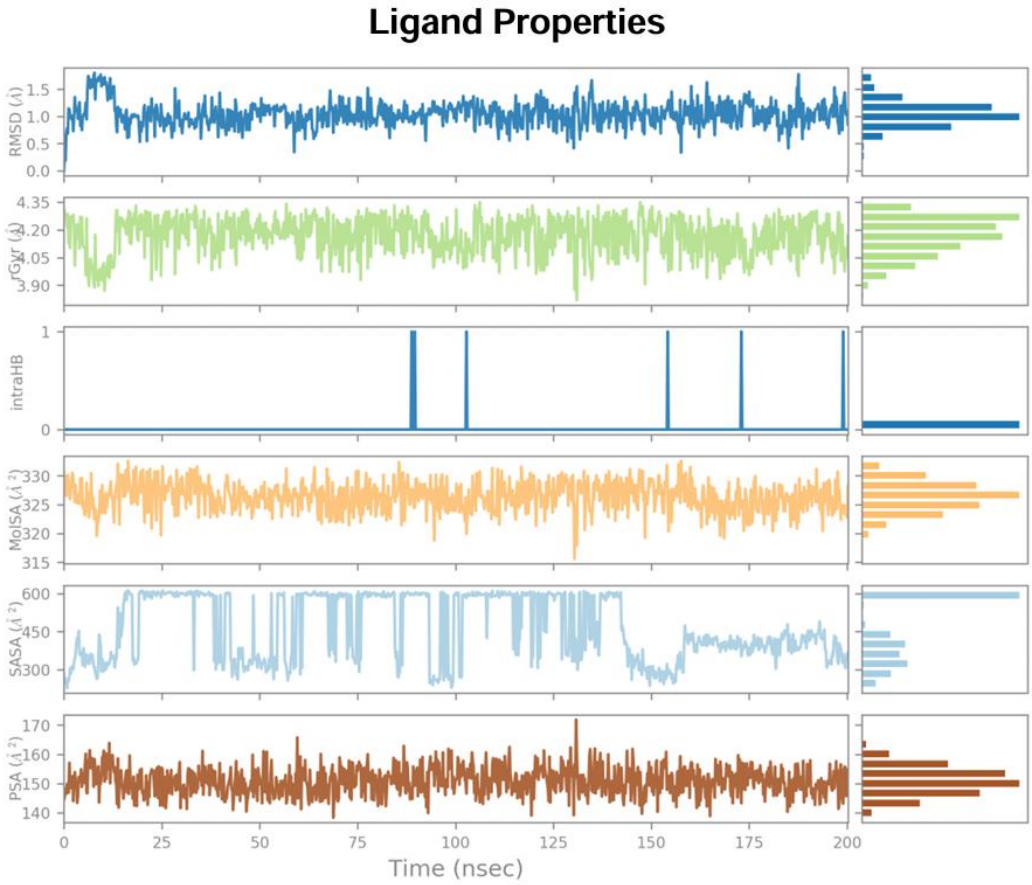

Figure S21: Molecular dynamics ligand properties trajectory analysis of NF-κB (PDB ID: 4Q3J) in complex with D4Z over 200 ns. (Top to Bottom) Ligand RMSD monitoring confirming system equilibration and stable binding; ligand properties (rGyr, intraHB, MolSA, SASA, PSA) confirming conformational compactness; and protein–ligand contact histograms with timeline persistence mapping the predicted high-occupancy interaction network.

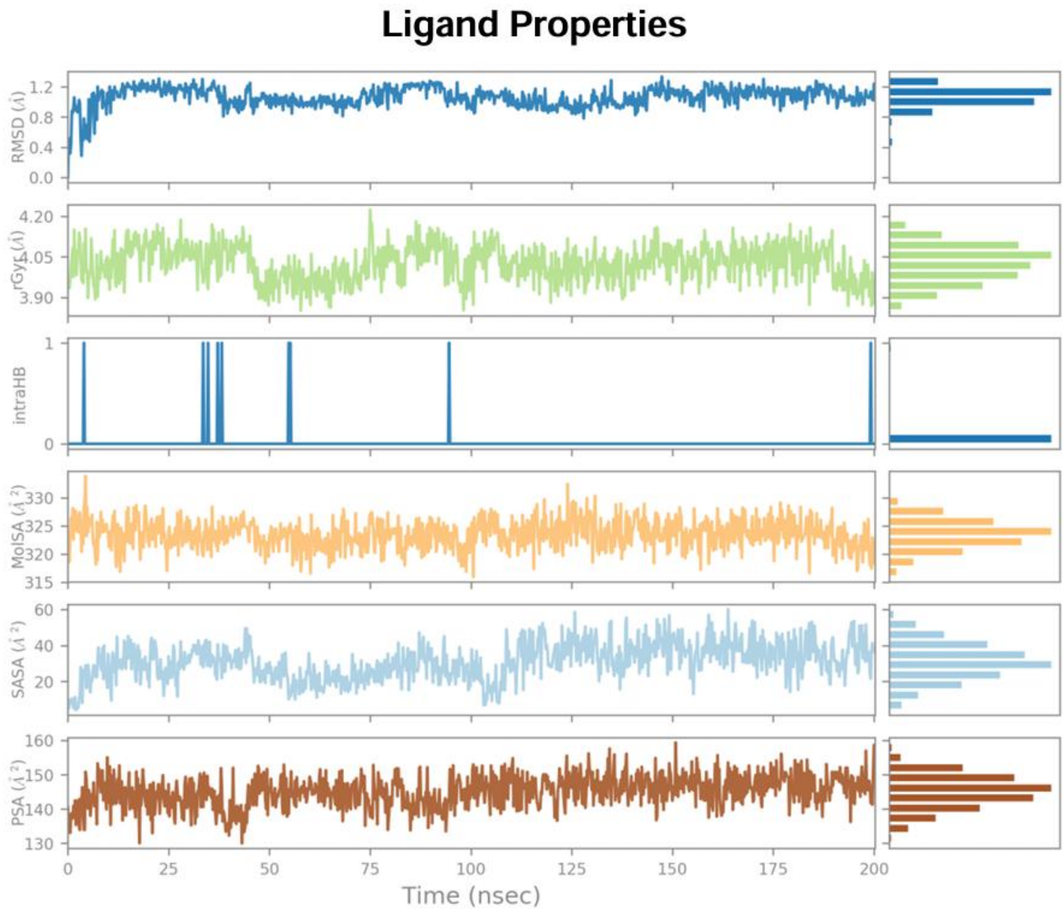

Figure S22: Molecular dynamics ligand properties trajectory analysis of NLRP3 (PDB ID: 5IRM) in complex with D4Z over 200 ns. (Top to Bottom) Ligand RMSD monitoring confirming system equilibration and stable binding; ligand properties (rGyr, intraHB, MolSA, SASA, PSA) confirming conformational compactness; and protein–ligand contact histograms with timeline persistence mapping the predicted high-occupancy interaction network.
